# DNA methylation from a Type I restriction modification system influences gene expression and virulence in *Streptococcus pyogenes*

**DOI:** 10.1371/journal.ppat.1007841

**Published:** 2019-06-17

**Authors:** Taylor M. Nye, Kristin M. Jacob, Elena K. Holley, Juan M. Nevarez, Suzanne Dawid, Lyle A. Simmons, Michael E. Watson

**Affiliations:** 1 Department of Molecular, Cellular, and Developmental Biology, University of Michigan, Ann Arbor, MI, United States of America; 2 Division of Pediatric Infectious Diseases, Department of Pediatrics and Communicable Diseases, University of Michigan, Ann Arbor, MI, United States of America; Boston Children's Hospital, UNITED STATES

## Abstract

DNA methylation is pervasive across all domains of life. In bacteria, the presence of N6-methyladenosine (m6A) has been detected among diverse species, yet the contribution of m6A to the regulation of gene expression is unclear in many organisms. Here we investigated the impact of DNA methylation on gene expression and virulence within the human pathogen *Streptococcus pyogenes*, or Group A Streptococcus. Single Molecule Real-Time sequencing and subsequent methylation analysis identified 412 putative m6A sites throughout the 1.8 Mb genome. Deletion of the Restriction, Specificity, and Methylation gene subunits (**Δ**RSM strain) of a putative Type I restriction modification system lost all detectable m6A at the recognition sites and failed to prevent transformation with foreign-methylated DNA. RNA-sequencing identified 20 genes out of 1,895 predicted coding regions with significantly different gene expression. All of the differentially expressed genes were down regulated in the **Δ**RSM strain relative to the parent strain. Importantly, we found that the presence of m6A DNA modifications affected expression of Mga, a master transcriptional regulator for multiple virulence genes, surface adhesins, and immune-evasion factors in *S*. *pyogenes*. Using a murine subcutaneous infection model, mice infected with the **Δ**RSM strain exhibited an enhanced host immune response with larger skin lesions and increased levels of pro-inflammatory cytokines compared to mice infected with the parent or complemented mutant strains, suggesting alterations in m6A methylation influence virulence. Further, we found that the **Δ**RSM strain showed poor survival within human neutrophils and reduced adherence to human epithelial cells. These results demonstrate that, in addition to restriction of foreign DNA, gram-positive bacteria also use restriction modification systems to regulate the expression of gene networks important for virulence.

## Author summary

DNA methylation is common among many bacterial species, yet the contribution of DNA methylation to the regulation of gene expression is unclear outside of a limited number of gram-negative species. We characterized sites of DNA methylation throughout the genome of the gram-positive pathogen *Streptococcus pyogenes* or Group A Streptococcus. We determined that the gene products of a functional restriction modification system are responsible for genome-wide m6A. The mutant strain lacking DNA methylation showed altered gene expression compared to the parent strain, with several genes important for causing human disease down regulated. Furthermore, we showed that the mutant strain lacking DNA methylation exhibited altered virulence properties compared to the parent strain using various models of pathogenesis. The mutant strain was attenuated for both survival within human neutrophils and adherence to human epithelial cells, and was unable to suppress the host immune response in a murine subcutaneous infection model. Together, these results show that bacterial m6A contributes to differential gene expression and influences the ability of Group A Streptococcus to cause disease. DNA methylation is a conserved feature among bacteria and may represent a potential target for intervention in effort to interfere with the ability of bacteria to cause human disease.

## Introduction

DNA methylation has been shown to regulate diverse pathways across all domains of life [1]. In eukaryotes, cytosine methylation regulates developmental gene expression and aberrant DNA methylation patterns have been implicated in many disease states, including cancer [2, 3]. Although studied in a limited number of prokaryotic organisms, DNA methylation has been implicated in a myriad of cellular processes, including protection from the invasion of foreign DNA, cell cycle regulation, DNA mismatch repair, and the regulation of gene expression [4]. It was recently shown that within the genomes of over 200 prokaryotes surveyed greater than 90% contained N6-methyladenosine (m6A), N4-methylcytosine (m4C), or 5-methylcytosine modifications (m5C) [5]. These results demonstrate that DNA methylation among prokaryotes is more pervasive than originally anticipated. What remains uncertain is if DNA methylation imparts any regulatory controls influencing virulence properties or other phenotypes amongst the array of diverse prokaryotic species.

DNA methylation in bacteria has been well characterized in the context of restriction modification (RM) systems [4, 5]. RM systems are a mechanism of bacterial host defense to prevent the invasion of foreign DNA. RM systems are generally comprised of a site-specific restriction endonuclease (REase), methyltransferase (MTase), and, in some cases, a specificity subunit that together form a protein complex that cleaves foreign DNA after it enters the cell. Methylation of the host DNA at the same recognition site serves to safeguard the host chromosome from cleavage. In addition to RM systems, DNA can also be methylated by orphan MTases. Orphan MTases methylate DNA in site-specific sequences and lack an active cognate endonuclease [5, 6]. In bacteria, the two most well studied orphan MTases are *Escherichia coli* DNA adenosine methyltransferase (Dam) and *Caulobacter crescentus* cell cycle regulated methyltransferase (CcrM) [5, 6]. Site-specific DNA methylation by Dam and CcrM has been shown to regulate DNA mismatch repair, cell cycle progression, origin sequestration, and gene expression, demonstrating that DNA methylation imparts critical regulatory functions [6].

Despite the importance of RM systems and orphan MTases, the lack of genome-wide detection tools has hindered the identification of DNA base modifications and characterization of the physiological consequences resulting from MTase inactivation in bacteria. The use of methylation-sensitive restriction endonucleases to identify sites of DNA base modifications is limited by the sequence specificity of the recognition site, potentially missing many base modifications that could occur outside of a particular sequence context ([5] and references therein). While bisulfite sequencing allows for genome-wide detection of m5C in sequence specific-contexts, no such genome-wide detection tool has been available for the detection of m6A or m4C until the recent advent of Pacific Biosciences (PacBio) Single Molecule Real-Time (SMRT) sequencing platform [7–11]. SMRT sequencing relies on differences in DNA polymerase kinetics to detect base modifications in the template strand in a sequence-context specific manner without *a priori* knowledge of the modification.

Our group previously used the PacBio SMRT sequencing platform to complete whole genome sequencing and reference genome assembly of two strains of the bacterial human pathogen *Streptococcus pyogenes*, or Group A Streptococcus (GAS) [12, 13]. *S*. *pyogenes* causes a wide variety of human infections, ranging from the relatively common streptococcal pharyngitis and cellulitis to the relatively uncommon, but severe, streptococcal toxic shock syndrome and necrotizing fasciitis, which have high morbidity and mortality rates [14–16]. *S*. *pyogenes* is a model bacterial pathogen, not only for the infections it produces, but also for the great diversity of toxins and virulence factors expressed by the organism and the highly complex nature of regulatory mechanisms employed to control virulence factor expression [14, 16–18]. Indeed, *S*. *pyogenes* utilizes over 30 recognized transcriptional regulatory proteins and 13 two-component regulatory systems to coordinate virulence factor expression in response to varying environmental signals (e.g., carbohydrate availability, temperature, pH, oxygen tension, salt concentrations, osmolality, etc.), growth phase, intracellular metabolite concentrations, and signaling pheromones involved in quorum sensing [17, 18]. DNA methylation has not been previously investigated as a significant mechanism influencing virulence factor expression within *S*. *pyogenes*, and DNA methylation may represent an unrecognized target for therapeutic intervention to help prevent or treat severe streptococcal disease.

In this study, we show that in *S*. *pyogenes* strain MEW123, a representative derivative of a serotype M28 clinical pharyngitis isolate, the active Type I RM system SpyMEW123I is responsible for the bipartite m6A motif identified throughout the genome. We show that deletion of the RM system and subsequent loss of m6A from *S*. *pyogenes* results in the down regulation of a distinct set of operons involved in streptococcal virulence. Importantly, our study shows that methylation by a Type I RM system correlates with differential expression of Mga, a major transcriptional regulator of multiple virulence factors, surface adhesins, and immune evasion factors in *S*. *pyogenes*. The results presented here demonstrate that RM systems can integrate their methylation signal to influence the expression of gene networks important for bacterial virulence.

## Results

### SMRT sequencing and methylation analysis identifies m6A modifications in a bipartite recognition sequence in the *S*. *pyogenes* genome

**Fig 1:**
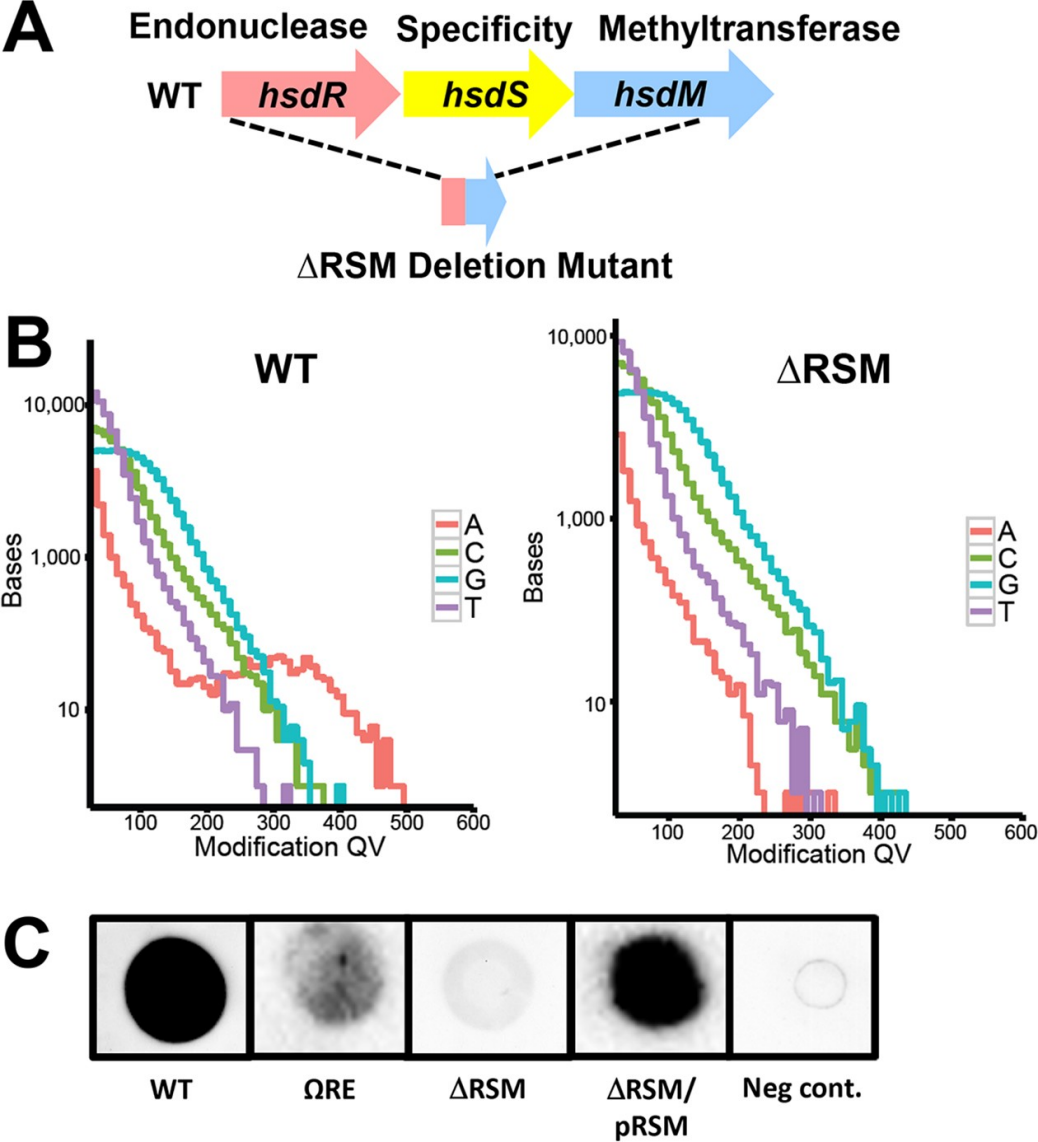
M.SpyMEW123I dependent m6A modifications in the *S*. *pyogenes* genome. A) Genomic organization of the SpyMEW123I Type I RM system gene cluster, *hsdRSM*, in strain MEW123 (WT) and the MEW513 in-frame deletion mutant (**Δ**RSM). B) Detection of genomic m6A base modifications (red line) in the MEW123 genome or the **Δ**RSM genome via PacBio SMRT sequencing. Modification quality values (modQVs) are indicated on the x-axis and the number of bases is indicated on the y-axis. ModQVs indicate if the polymerase kinetics at a position differs from the expected background, where a modQV of 30 corresponds to a p-value of 0.001. C) Dot blot with α-m6A antibody on genomic DNA isolated from the following strains: MEW123 (WT), restriction endonuclease *hsdR* antibiotic cassette-disruption mutant MEW489 (ΩRE), in-frame deletion of the *hsdRSM* gene cluster (**Δ**RSM), the **Δ**RSM strain complemented with plasmid-encoded *hsdRSM* (**Δ**RSM/pRSM), and unmodified DNA oligonucleotides serving as a negative control (Neg cont.) (500 ng DNA per spot).

Previously we completed whole genome assembly using PacBio SMRT sequencing with *S*. *pyogenes* strain MEW123, a representative serotype M28 isolate used by our group to investigate streptococcal mucosal colonization [12] (for strain list refer to Table 1). To begin our investigation, we performed methylation analysis of the SMRT sequencing data. We identified m6A DNA base modifications in the MEW123 genome at the consensus sequence 5' GCANNNNNTTYG and its corresponding partner motif 5' CRAANNNNNNTGC, consistent with m6A modification motifs previously reported by Blow *et al*. (Table 2) [5]. Within the MEW123 genome, 412 occurrences of each m6A site within the bipartite recognition motif were identified; the majority occurred in predicted coding (92%) and intergenic (6%) regions of the MEW123 genome. The bipartite recognition motif is characteristic of Type I RM systems, which are typically comprised of three separate subunits, including a restriction endonuclease, a specificity subunit, and a methyltransferase subunit, that act together as a single protein complex and typically act at large distances from the methylation site. The RM system annotation pipeline used in Blow *et al*. identified the putative Type I restriction modification system, annotated as SpyMEW123I, consisting of a three-gene cluster with separate restriction endonuclease (*hsdR*), specificity (*hsdS*), and methyltransferase (*hsdM*) genes, as a predicted match for modification of the identified m6A motif in *S*. *pyogenes* [5, 19] (Fig 1A and Fig 1B). This three-gene cluster exhibits high amino acid sequence homology to the Type I RM system identified in *S*. *pyogenes* SF370 at Spy_1904 (*hsdR*), Spy_1905 (*hsdS*), and Spy_1906 (*hsdM*), with 99%, 87%, and 99% identity, respectively [20]. This Type I RM system is present in virtually all sequenced *S*. *pyogenes* strains to date, with rare exception reported in some *emm1* strains from Japan with spontaneous deletion of a two-component regulatory system and the adjacent Type I RM system [21]. Notably, we did not detect the 5mC modifications at C^m^CNGG reported by Euler *et al*. in our PacBio SMRT sequencing results, which is not surprising given the MTase, M.SpyI, is absent from the *S*. *pyogenes* M28 serotype [22]. The REase and MTase activities of SpyMEW123I are annotated as R.SpyMEW123I and M.SpyMEW123I, respectively.

**Table 1:** Strains used in this study.

Strain/Plasmids	Description	Source
**Strains**		
*Escherichia coli*		
DH5α	Standard cloning vector, *recA1 endA1 hsdR17*	Invitrogen
*Streptococcus pyogenes*		
MEW123	Streptomycin-resistant clone of pediatric throat isolate, serotype M28	[12]
MEW380	MEW123 transformed by plasmid pIL09 to insertionally-inactivate scpA gene by spectinomycin resistance marker (Ω*scpA*)	This study
MEW409	MEW123 transformed with plasmid pIL03 to insertionally-inactivate M protein, *emm* gene, by spectinomycin resistance marker (Ω*emm28*)	This study
MEW480	MEW123 with in-frame deletion of *mga* (**Δ***mga*) by allelic exchange after transformation with plasmid pIL01	This study
MEW489	MEW123 transformed with plasmid pKJ19 to insertionally-inactivate restriction endonuclease with spectinomycin resistance marker (ΩRE)	This study
MEW513	MEW123 with restriction-modification system in-frame deletion (**Δ**RSM) by allelic exchange after transformation with pKJ24	This study
MEW552	MEW513 with RSM operon complemented *in trans* on plasmid pEH01 (**Δ**RSM/pRSM)	This study
HSC5	Serotype M14 reference strain	[23]
HSC5 Ωemm	HSC5 mutant with the M protein, *emm14* gene, disrupted by spectinomycin resistance marker (Ω*emm14*)	[24]
**Plasmids**		
pJRS233	Low-copy *E*. *coli* to *S*. *pyogenes* temperature-sensitive vector for allelic replacement (erythromycin-resistant, Erm^R^)	[25]
pGCP213	High-copy *E*. *coli* to *S*. *pyogenes* temperature-sensitive vector for allelic replacement (Erm^R^)	[26]
pJoy3	*E*. *coli* to *S*. *pyogenes* shuttle vector (chloramphenicol-resistant, Chlor^R^)	[27]
pSpc18	Integration vector containing aad9 (spectinomycin resistance gene from *Enterococcus faecalis*) (Spc^R^)	[28]
pIL01	pJRS233 with in-frame deletion of *mga* (Erm^R^)	This study
pIL03	pSpc18 with *emm28* gene fragment disruption (Spc^R^)	This study
pIL09	pSpc18 with *scpA* gene fragment disruption (Spc^R^)	This study
pKJ19	pSpc18 with restriction endonuclease gene fragment disruption (Spc^R^)	This study
pKJ24	pGCP213 with in-frame deletion of restriction-modification gene cluster (Erm^R^)	This study
pEH01	pJoy3 with intact RSM operon cloned for complementation (Chlor^R^)	This study

**Table 2:** Motif analysis of modified bases from PacBio SMRT sequencing in wild type *S*. *pyogenes* strain MEW123.

Motif*	Type	Motifs in genome	Mean modQV	Mean coverage
GC**A**NNNNNNNTTYG	m6A	412	332.64	245.02
CRA**A**NNNNNNNTGC	m6A	412	282.18	237.32
**T**HTWGAAGA	unknown	410	44.74	240.57
**A**NDYVGCAD	m6A	3502	86.17	241.02
**T**NRRDDDG	unknown	34390	44.88	235.68
**T**NNNDNNH	unknown	746710	47.81	235.15
**T**HRGCNTWNH	unknown	3107	43.39	237.84
**A**GNNAVNW	m6A	32122	78.13	239.29
**T**NNNCRV	unknown	77468	45.11	235.03
V**A**HNBAVYW	m6A	27467	76.34	242.45
**T**HNNDVNG	unknown	96451	43.43	237.89

*modified base is bolded and underlined

### M.SpyMEW123I is responsible for m6A modifications in the *S*. *pyogenes* genome

To determine if the SpyMEW123I RM system was responsible for the observed m6A modifications in strain MEW123, an in-frame deletion mutation was constructed using a plasmid vector designed for allelic replacement (pGCP213) as previously described [26] (Table 1 and Fig 1A). Approximately 95% of the three-gene sequence encoding the *hsd**R*, *hsd**S*, and *hsd**M* genes was deleted producing strain MEW513 (referred to as **Δ**RSM); the in-frame deletion was confirmed by PCR amplification and Sanger DNA sequencing (Table 1). Growth of the MEW123 parent strain, referred to as wild-type (WT) and the **Δ**RSM mutant were not significantly different in rate or final growth density when measured in either the nutrient rich Todd-Hewitt medium with 0.2% yeast extract (THY broth) or the low-carbohydrate C-medium (S1 Fig). To confirm a reduction in m6A base modifications and to determine the sequence context lacking m6A base modifications in the **Δ**RSM strain, genomic DNA was isolated and sequenced via PacBio SMRT sequencing. Modification analysis showed loss of detectable m6A base modifications at 5' GCANNNNNTTYG and 5' CRAANNNNNNTGC sites, demonstrating that streptococci with a SpyMEW123I deletion no longer have m6A DNA base modifications at the consensus sequence identified in the WT strain (Fig 1B, Table 3). A number of additional methylation events were identified in MEW513; however, these occurred at far lower frequencies compared to the modifications at the consensus sequences in the parent strain and the quality of the read scores (Mod QV) were low compared to the RSM-dependent modifications. Based on these low quality read scores, we feel it is unlikely that these additional modifications reflect compensatory methylation events. Furthermore, SMRT sequencing of the MEW513 genome did not identify any unforeseen mutations outside of the in-frame deletion within *hsdRSM* that we anticipated.

**Table 3:** Motif analysis of modified bases from PacBio SMRT sequencing in ΔRSM strain MEW513.

Motif*	Type	Motifs in genome	Mean modQV	Mean coverage
**T**YTWGARGR	unknown	701	46.56	263.00
D**A**GKBANYW	m6A	5826	88.94	256.78
**A**NNYRGYA	m6A	8371	85.54	259.83
G**A**HBBAACA	m6A	499	125.47	268.90
**T**NNNDNNH	unknown	746710	49.02	251.20
**T**NRRDDDG	unknown	34390	45.12	251.97
**T**HRGCNTH	unknown	5464	43.69	253.61
**T**NNNCRV	unknown	77468	45.81	251.94
D**T**NRVCBNHNH	unknown	29343	44.13	251.93
**A**HSBAMYW	m6A	9198	75.01	265.11
**T**HNNDVNG	unknown	96451	44.13	256.11

*modified base is bolded and underlined

To further confirm that the MTase component of the RSM gene cluster, M.SpyMEW123I, was indeed responsible for producing m6A DNA modifications, genomic DNA was harvested from the WT and the **Δ**RSM strain and spotted onto a nitrocellulose membrane for immunodetection using an α-m6A antibody. We found that the α-m6A signal was substantially reduced in genomic DNA blots from the **Δ**RSM strain compared to the WT parent, suggesting a significant and near complete reduction in m6A base modifications in the **Δ**RSM strain (Fig 1C). Complementation *in trans* of the **Δ**RSM mutant with a plasmid encoded copy of the three gene cluster (*hsdRSM*) produced strain MEW552 (referred to as **Δ**RSM/pRSM) and successfully restored detection of the α-m6A signal to levels comparable to the WT strain (Fig 1C). These results demonstrate that the MTase activity of SpyMEW123I is responsible for base modifications at 5' GCANNNNNTTYG and 5' CRAANNNNNNTGC sites *in vivo*.

### The SpyMEW123I RM system influences *S*. *pyogenes* transformation efficiency demonstrating functional restriction of foreign DNA acceptance

**Fig 2:**
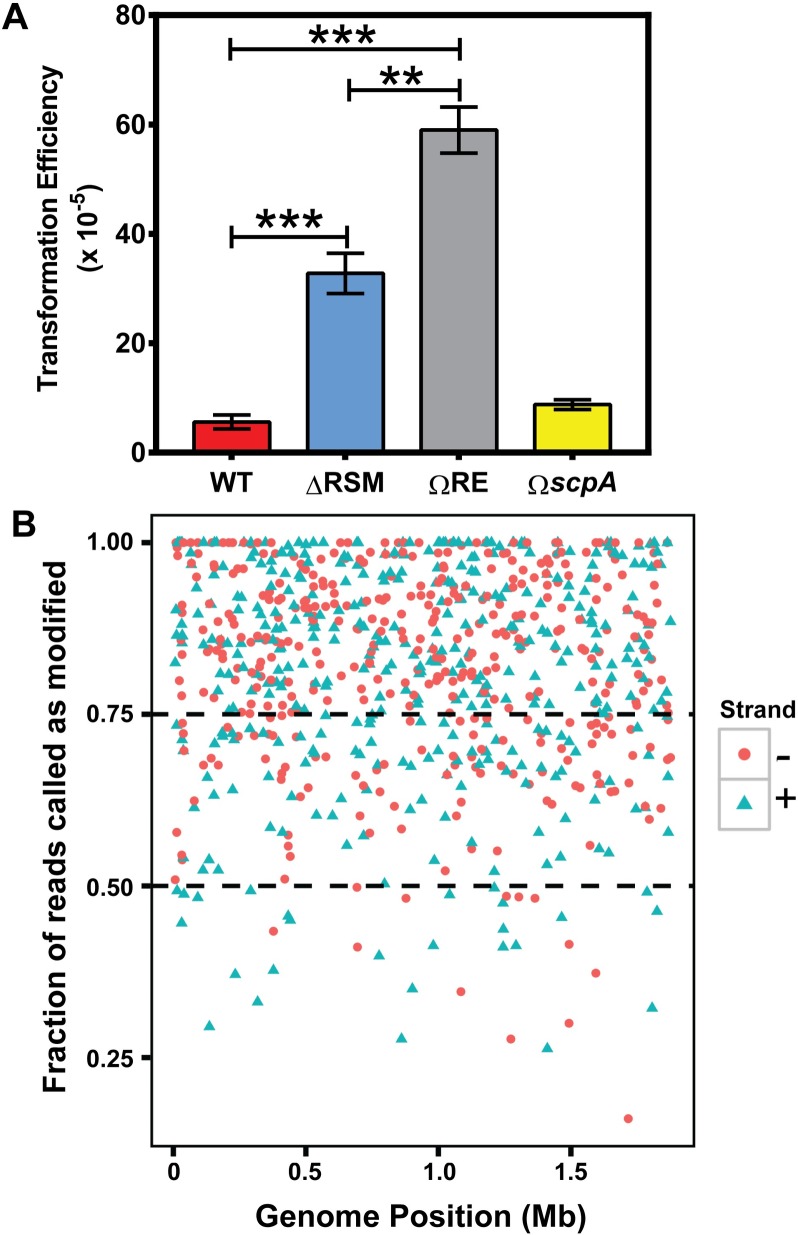
The Type I RM system, SpyMEW123I, is functional for endonuclease activity and influences transformation efficiency. A) Transformation efficiencies of WT (MEW123), the *hsdRSM* in-frame deletion (**Δ**RSM, MEW513), the *hsdR* single-gene disrupted mutant (ΩRE, MEW489), and the *scpA* insertional inactivation mutant (Ω*scpA*, MEW380). A Mann-Whitney U test was used to identify significant differences between groups for transformation efficiency; ** *P* < 0.01, *** *P* < 0.001, n = 3–7 replicates per point. B) Each SpyMEW123I RM site is represented on the plot where the position that the site occurs in the genome (from 0 Mb -1.8 Mb) is represented on the x-axis. The fraction of reads called as methylated from PacBio SMRT sequencing for each RM site is represented on the y-axis. RM sites that occur on the “+” and “-” strand of the DNA duplex are represented by green triangles and red dots, respectively.

Deletion of the three-gene cluster, *hsdRSM*, containing the predicted endonuclease, specificity, and methylation gene subunits abolished m6A base modifications in the **Δ**RSM mutant strain. In Type I RM systems, DNA cleavage is dependent on the MTase and specificity subunits, in addition to the REase subunits which are often independently regulated by a separate promoter [29]. Fully unmethylated recognition motifs induce REase activity that results in DNA cleavage typically between two fully unmethylated motifs at sites distant from the recognition sequence; this distance may range from 40 base pairs to several kilobases away from the RM site. Type I MTases can function to add m6A *de novo* on fully unmethylated DNA or act as maintenance MTases at hemi-methylated recognition sites [29–31]. Additional mechanisms also protect DNA from restriction, including proteolysis of the REase subunits or protection by DNA binding proteins that can protect unmethylated sites from cleavage in the host chromosome [32]. To establish the functionality of the REase component of SpyMEW123I, a transformation efficiency assay was performed using pJoy3 plasmid DNA methylated in an *E*. *coli* host (Table 1). This 6.3 kb plasmid contains eight predicted Dam MTase RM sites (5' GATC) and is delivered in its native double-stranded circular form via electroporation into electrocompetent *S*. *pyogenes* where the plasmid is maintained and replicates extrachromosomally [27]. In addition to testing the effect of deleting the entire *hsdRSM* gene cluster in the **Δ**RSM mutant strain, we constructed an additional strain derivative of MEW123 with a spectinomycin-resistance cassette disrupting the *hsdR* REase gene subunit alone producing strain MEW489 (referred to as ΩRE, Table 1). If the SpyMEW123I RM system has true restriction enzyme activity to foreign-modified DNA, then we would expect that inactivating the *hsdR* gene subunit, either individually or within the entire RSM gene cluster, would enhance the transformation efficiency of the plasmid. Indeed, we found that the rates of transformation with foreign-methylated plasmid DNA increased significantly for both the **Δ**RSM mutant and the ΩRE mutant strains compared to the WT parent strain, providing evidence that the restriction endonuclease component of SpyMEW123I is active and functional (Fig 2A). We were unable to compare our complementation strain **Δ**RSM/pRSM for transformation efficiency as this strain already carries the pJoy3 plasmid encoding the *hsdRSM* gene cluster. As a control, we undertook transformation of a MEW123 mutant in the gene encoding the C5a peptidase, *scpA* (strain 489 or Ω*scpA*), as mutants in this gene would not be expected to show enhanced transformation efficiency; as expected, the transformation efficiency of Ω*scpA* was not significantly different than the WT (Fig 2A). Interestingly, inactivation of the endonuclease subunit *hsdR* alone in the ΩRE mutant strain conferred significantly greater transformation efficiency than that observed in the **Δ**RSM mutant (Fig 2A). In many Type I RM systems the restriction subunit is generally under control of a separate promoter than the specificity and methylation subunits in the RSM gene cluster [29]. We found that the α-m6A signal generated by dot blot of genomic DNA from the ΩRE strain was intermediate in intensity between the WT and **Δ**RSM strains (Fig 1C). This result suggests that the methyltransferase subunit was still functional in the ΩRE strain, but that there may have been some degree of polar effect from the spectinomycin-resistance cassette used to inactivate *hsdR* that was reducing transcription of the *hsdS* and *hsdM* gene products compared to WT levels. We speculate that the residual functional activities of the specificity and methyltransferase subunits in the ΩRE mutant strain, even though less than WT levels, may have conferred additional stability to the incoming foreign-methylated plasmid DNA, possibly offering protection from other minor endonucleases, thereby enhancing overall transformation efficiency.

### Variation in m6A base modification occurrence at RM recognition sites identified by SMRT sequencing in the *S*. *pyogenes* genome

As discussed above, the MTase activity of Type I RM systems may function to maintain the state of hemi-methylated or fully methylated DNA, whereas REase cleavage only occurs on fully unmethylated DNA. Thus, RM sites can exist in the genome in a hemi-methylated state while still conferring protection from digestion [29]. Having shown that SpyMEW123I functions as an active RM system, we sought to establish the fraction of reads that were called as methylated at each recognition site. Our analysis of sequencing data from WT *S*. *pyogenes* MEW123 found substantial variation in the fraction of sequencing reads modified at RM sites (Fig 2B). The fraction of reads called as modified at a given RM site did not appear to be dependent on orientation or genome position. Of the m6A modifications called at RM sites, 4.9% of sites were called as m6A modified in less than 50% of sequencing reads, 23.7% of sites were called as modified in between 50–75% of sequencing reads, and finally 71.4% of sites were called as modified at greater than 75% of aligned reads. Previous studies have also reported heterogeneity in the frequency of SMRT sequencing reads with base modifications; it has been hypothesized that these differences are due to timing in DNA replication and subsequent methylation [33–35]. Whether there is a temporal component accounting for the heterogeneity in m6A DNA modifications, and whether this impacts other functions of m6A modifications, such as in influencing gene transcription in *S*. *pyogenes*, is unknown. Given the heterogeneity observed in the fraction of reads called as m6A methylated, we hypothesized that m6A modifications produced by the SpyMEW123I RM system might have additional functions outside of host protection from foreign DNA prompting the experiments below.

### RNA-sequencing shows that deletion of the SpyMEW123I RM system results in the down regulation of several transcripts involved in streptococcal immune evasion and adherence

**Fig 3:**
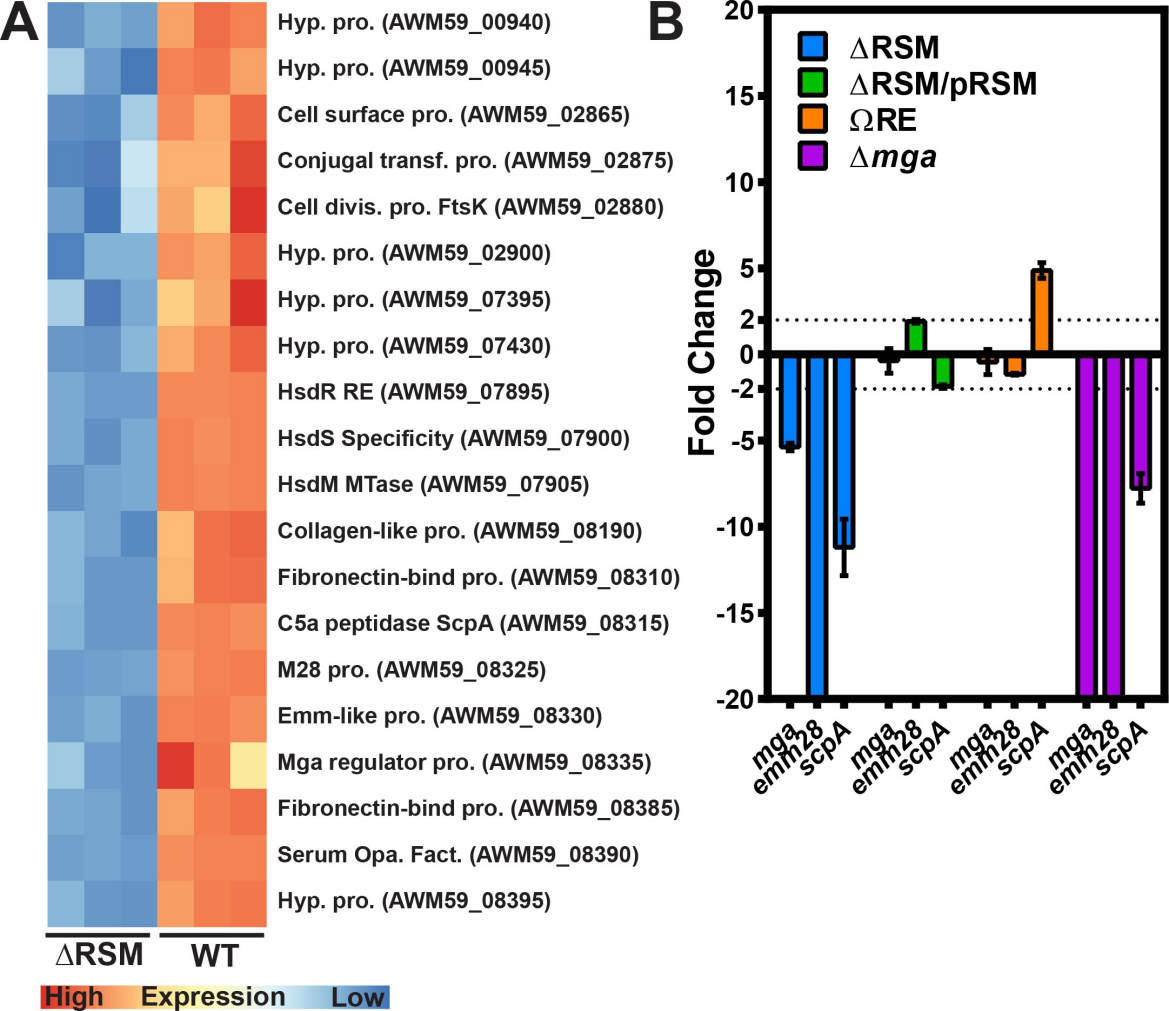
Gene transcripts involved in immune evasion and adherence are down regulated in the ΔRSM strain compared to WT. A) Heatmap of differentially expressed genes between WT and **Δ**RSM strains. Replicates in triplicate are represented on the x-axis and published/putative gene functions are on the y-axis with gene references to the MEW123 genome. Relative expression is compared row-wise with more highly expressed replicates in red. B) Verification of RNA-Seq data using qRT-PCR with individual primer sets shown on the x-axis for the **Δ**RSM mutant (blue), complemented mutant strain **Δ**RSM/pRSM (green), *hsdR* single gene mutant ΩRE (orange), and the **Δ***mga* mutant (purple). The y-axis indicates Relative Transcript Levels for individual transcripts compared to *recA* reference transcript. Each gene transcript was analyzed in triplicate. Shown is fold change compared to WT expression, with genes showing greater than two-fold change as significant. Genes extending lower than the x-axis were down-regulated several hundred-fold.

In addition to functioning in RM systems, m6A base modifications from orphan MTases have been shown to function in cell cycle regulation, DNA mismatch repair, and the regulation of transcription [4]. In the pathogenic *Escherichia coli* serotype O104:H4 strain C227-11 associated with hemolytic uremic syndrome, deletion of the ϕStx104 RM system results in the differential expression of over 38% of the genes, including genes involved in motility, cell projection, and cation transport [33]. Mismatch repair is not coupled to methylation in *S*. *pyogenes* or most other gram-positive bacteria [36]. Therefore, we asked if m6A originating from the SpyMEW123I RM system in *S*. *pyogenes* might have additional functions outside of host defense from foreign DNA. We isolated RNA from streptococcal cells during mid-exponential growth phase in C media broth culture from WT and **Δ**RSM strains followed by RNA-sequencing. The results of the differential expression analysis showed that 20 genes were differentially expressed in the **Δ**RSM strain compared to WT (adjusted p. val < 0.05, log_2_ fold change >1, data set available at NCBI repository). Interestingly, all 20 genes were down regulated in **Δ**RSM relative to WT suggesting a common regulatory mechanism (Fig 3A and 3B, Table 4). The three genes (*hsdRSM*) of the SpyMEW123I RM gene cluster showed the greatest log_2_ fold change in expression of -10.8, -10.7, and -11.7, respectively, which was expected because these genes were deleted in the **Δ**RSM strain.

**Table 4:** Differentially expressed genes in ΔRSM strain compared to WT.

Cluster	MEW123 Locus	Fold-Change (Log2)	Ave Expr	adj.P.Val	Prob	Product Annotation
1	AWM59_00940	-3.88	1.98	0.00	1.00	hypothetical protein
1	AWM59_00945	-3.16	1.30	0.00	0.98	hypothetical protein
2	AWM59_02865	-1.14	6.80	0.01	0.89	cell surface protein
2	AWM59_02875	-1.12	3.04	0.05	0.45	conjugal transfer protein
2	AWM59_02880	-1.29	4.30	0.04	0.46	cell division protein (FtsK)
2	AWM59_02900	-1.14	2.87	0.01	0.87	hypothetical protein
3	AWM59_07395	-1.23	6.09	0.03	0.55	hypothetical protein
3	AWM59_07430	-4.12	5.14	0.00	1.00	hypothetical protein
4	AWM59_07895	-10.73	4.10	0.00	1.00	Endonuclease *hsdR*
4	AWM59_07900	-11.69	3.07	0.00	1.00	Specificity *hsdS*
4	AWM59_07905	-10.79	3.98	0.00	1.00	Methyltransferase *hsdM*
5	AWM59_08190	-5.60	5.26	0.00	0.99	Collagen-like surface protein A (SclA)
5	AWM59_08310	-2.38	6.45	0.00	0.99	LPXTG anchor domain surface protein
5	AWM59_08315	-4.32	5.53	0.00	1.00	peptidase C5 (ScpA)
5	AWM59_08325	-8.65	5.31	0.00	1.00	M28 protein (M28)
5	AWM59_08330	-8.01	6.54	0.00	1.00	emm-like protein
5	AWM59_08335	-1.24	6.39	0.04	0.45	Trans-Acting Positive Regulator (Mga)
6	AWM59_08385	-4.57	6.93	0.00	1.00	fibronectin-binding protein (SfbX)
6	AWM59_08390	-5.11	7.31	0.00	1.00	Serum Opacity Factor (SOF)
6	AWM59_08395	-6.47	3.57	0.00	1.00	hypothetical protein

The majority of the differentially expressed genes are located in approximately 6 separate operons or gene clusters as indicated in Table 4. Interestingly, several of these gene groups are transcriptionally regulated, at least in large part, by activity of the multiple gene regulator protein, Mga [37–39]. During mid-exponential growth phase, Mga acts as a transcriptional activator to regulate a core set of virulence factors at the *mga* locus [37]. The *mga* locus consists of several components: a) the M protein (*emm* gene) a major surface protein involved in resistance to phagocytosis and intracellular killing by neutrophils and used to distinguish *S*. *pyogenes* isolates, b) a fibronectin-binding protein that binds host complement regulator factors, c) an *emm*-like protein that binds IgG and fibrinogen, d) the C5a peptidase (ScpA) which cleaves C5a chemotaxin, e) the *enn* protein that binds IgA, and f) the *mga* gene itself. All genes at the *mga* locus displayed log_2_ fold changes ranging from -1.2 to -8.7 in the **Δ**RSM strain relative to WT (Fig 3A). To confirm this differential expression, we again isolated total RNA from strains during mid-exponential growth phase in C media broth culture and performed quantitative RT-PCR for detection of transcripts *mga*, *emm28*, and *scpA* (Fig 3B). Consistent with the RNA-seq results, the qRT-PCR results showed that these genes were significantly down regulated in the **Δ**RSM strain, with approximately 5-fold to over 300-fold decreased expression in the **Δ**RSM strain relative to WT (Fig 3B). Complementation *in trans* in the **Δ**RSM/pRSM strain restored transcript expression patterns similar to WT values. Deletion of the *mga* gene produced qRT-PCR results in a similar trend to the **Δ**RSM strain for the examined transcripts, with significantly decreased detection of *emm28* and *scpA* transcripts; *mga* transcript was not detected in the **Δ***mga* strain (Fig 3B). Examination of these transcripts in the *hsdR* insertional inactivation mutant ΩRE showed transcript detection of *mga* and *emm28* comparable to WT levels, with detection of *scpA* transcript approximately four to five-fold of WT levels. This transcript pattern was very different than those of the **Δ**RSM and **Δ***mga* mutant strains and more similar to the WT pattern. Even though the spectinomycin resistance cassette insertion into *hsdR* may have produced some polar effect with slightly decreased methyltransferase activity as noted on the α-m6A dot blot (Fig 1C), it seems sufficient residual m6A base modifications persisted to not significantly disrupt gene expression (Fig 3B). Taken together, these results from RNA sequencing and qRT-PCR provide evidence that m6A base modifications correlate with patterns of differential gene expression in *S*. *pyogenes*, including those of several recognized virulence factors and major regulators of virulence gene expression.

### Disruption of m6A DNA modifications enhances the host inflammatory response to streptococcal infection in a murine subcutaneous ulcer model

**Fig 4:**
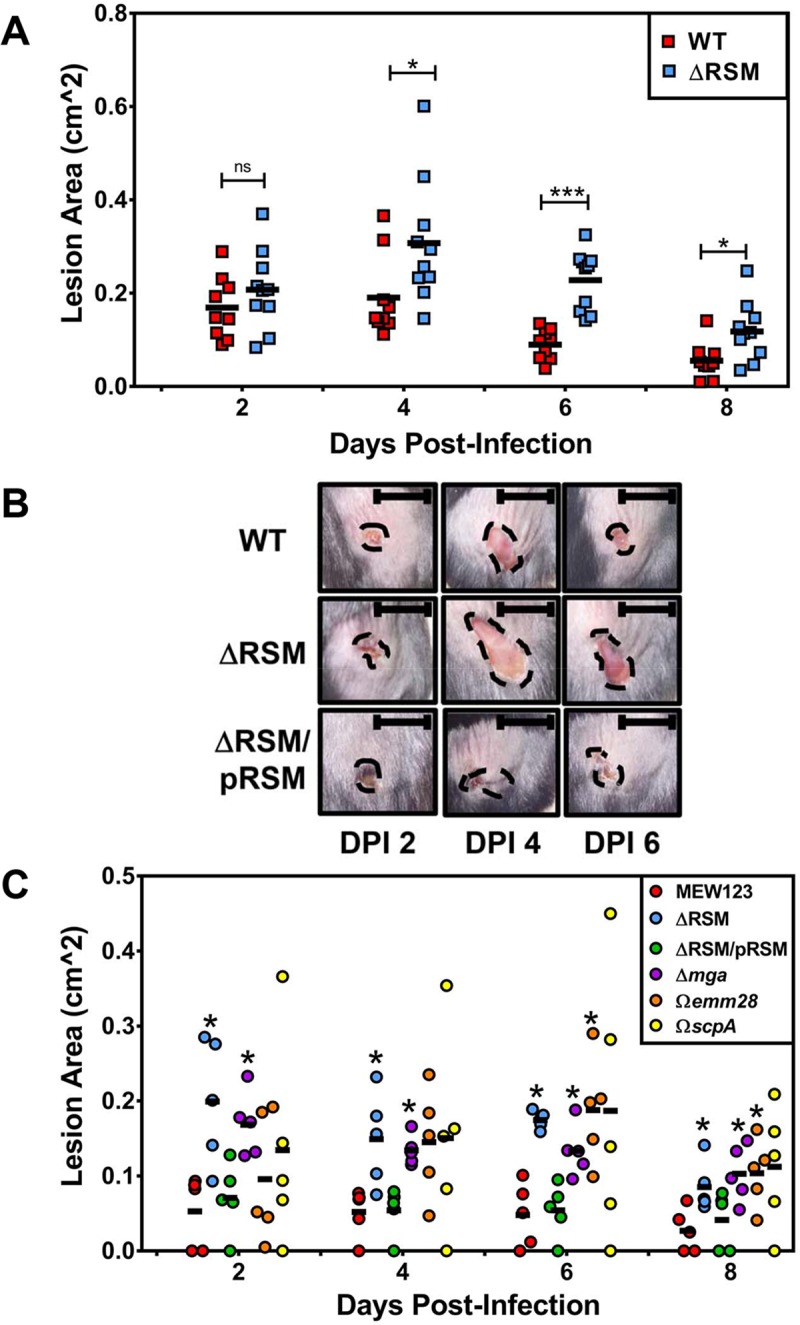
Deletion of the MEW123 RSM gene cluster is associated with larger skin lesion formation in a murine subcutaneous infection model. C57BL/6J mice were inoculated subcutaneously at the shaved flank with 1x10^7^ CFUs MEW123 (WT, red squares) or the **Δ**RSM mutant (blue squares) on Day 0. Lesions were photographed daily and lesion area was calculated using ImageJ software. A) Shown are individual mouse lesion area sizes with mean values (black bars) over Days Post-Infection (DPI). A Mann-Whitney U test was used to identify significant differences in lesion size between strains at each time point; ns, not significant, * *P* < 0.05, *** *P* < 0.001. B) Representative images of mice skin lesions over time at days 2-, 4-, and 6-days post-infection. Shown are skin lesions following infection with MEW123 (WT), MEW513 (**Δ**RSM), and the complemented strain MEW552 (**Δ**RSM/pRSM). Black bars are 1 cm for reference. Black dashed lines highlight the area of tissue injury. C) Shown are individual mouse lesion area sizes from a representative experiment with mean values (black bars) over Days Post-Infection. Shown are lesions from mice infected with MEW123 (WT), MEW513 (**Δ**RSM), the complemented strain MEW552 (**Δ**RSM/pRSM), the in-frame deletion of Mga, MEW480 (**Δ***mga*), the cassette-insertion disruption mutant of *emm28*, MEW409 (Ω*emm28*), and the cassette-insertion disruption mutant of *scpA*, MEW380 (Ω*scpA*). A Mann-Whitney U test was used to identify significant differences in lesion size between strains at each time point; * *P* < 0.05.

Given that the genes in the Mga regulon were significantly down regulated in the **Δ**RSM strain relative to WT, we were interested in determining the impact of disrupting m6A DNA modifications on *S*. *pyogenes* virulence using a murine subcutaneous infection model [40, 41]. C57BL/6J mice were inoculated at the shaved flank with 1 x 10^7^ CFUs of either MEW123 (WT) or the **Δ**RSM mutant strain and resulting skin ulcers were photographed daily for sizing the skin ulcer area. As shown in Fig 4A, there was no significant difference in skin lesion size at day two post-infection in comparison of the mice infected with either the WT or the **Δ**RSM strains. However, by three to four days post-infection, and for the remainder of the experiment, the skin lesions of mice infected with the **Δ**RSM strain were significantly larger than those of mice infected by the WT strain (Fig 4A). No strain caused a lethal infection among any of the mice with the 1 x 10^7^ CFU inoculum. Representative images of skin lesions for mice infected with the WT, the **Δ**RSM strain, and the complemented **Δ**RSM/pRSM strain over time are shown in Fig 4B, with skin lesions of mice infected with the **Δ**RSM strain notably larger on average at 4 and 6 days compared to those of mice infected with the WT or complemented strain. Complementation of the **Δ**RSM mutation *in trans* by strain MEW552 (**Δ**RSM/pRSM) produced murine skin lesions smaller than the **Δ**RSM mutant but not significantly different than the WT strain throughout the duration of the experiment (Fig 4C).

Skin lesion sizes reached a mean peak size at four to six days post infection. To determine if the difference in skin lesion size correlated with the concentration of viable streptococci at the site of infection, the skin lesions of mice were dissected and homogenized at day four post-infection to obtain viable CFU counts. Upon dissection, we made the observation that skin lesions from mice infected with the **Δ**RSM strain were grossly more purulent than lesions of mice infected by the WT and complemented **Δ**RSM/pRSM strains. The skin lesions contained on average CFU counts of approximately 1 x 10^6^ to 1 x 10^7^ CFUs; while there was a slight trend to higher CFU counts on day four post-infection for the **Δ**RSM streptococci compared to the WT and complemented strain CFUs, there were no statistically significant differences in CFU counts between these groups (S2 Fig). We noted that skin lesions of mice infected with the WT and complemented strain **Δ**RSM/pRSM strains seemed to heal more quickly than those of mice infected with the **Δ**RSM strain (Fig 4C).

**Fig 5:**
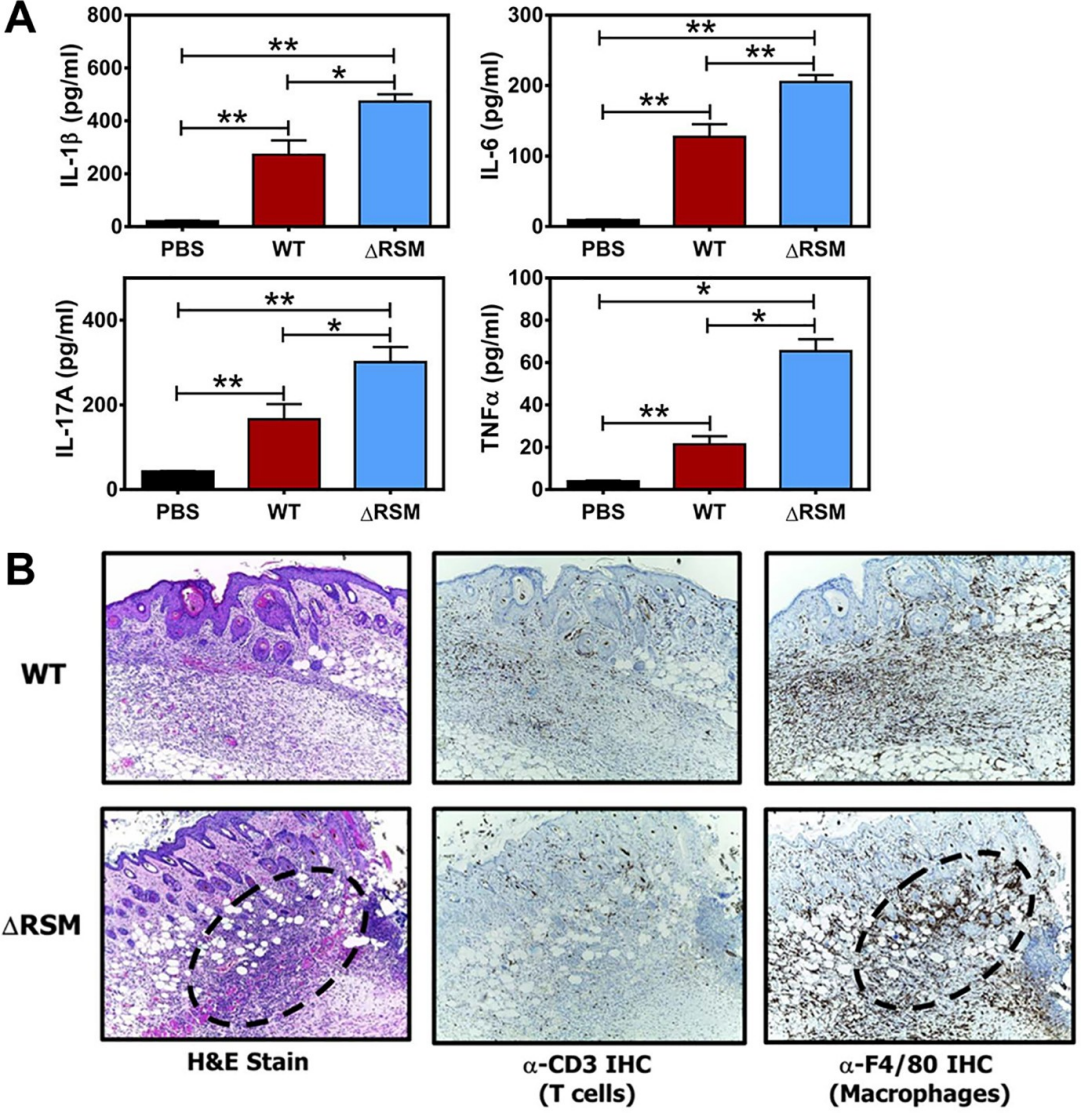
Deletion of the MEW123 RSM gene cluster increases the host inflammatory cytokine response in the murine subcutaneous infection model. C57BL/6J mice were inoculated subcutaneously at the shaved flank and resulting lesions were dissected on day 6 post-infection for cytokine analysis (panel A) and histology (panel B). A) Shown are ELISA results of homogenized murine skin biopsy specimens from mice previously inoculated with sterile phosphate buffered saline (PBS, black bars), 1x10^7^ CFUs MEW123 (WT, red bars), or 1x10^7^ CFUs of the **Δ**RSM mutant (**Δ**RSM, blue bars). Results are pooled from biopsies of 3 mice per group with mean and SEM cytokine concentrations. A Mann-Whitney U test was used to identify significant differences in cytokine concentration; ns, not significant, * *P* < 0.05, ** *P* < 0.01. B) Representative images at 10X magnification of skin biopsies from mice inoculated with MEW123 (WT, upper row), or the **Δ**RSM mutant (**Δ**RSM, bottom row). Slides were stained with hematoxylin and eosin (H&E) for general neutrophil and overall inflammatory response, or specifically by immunohistochemistry for T cells (α-CD3) or macrophages (α-F4/80). Focal areas of intense inflammation were outlined with dashed lines for comparison.

With the subcutaneous ulcer model, skin lesion size tends to correlate closely with the degree of the host immune response, with particular regards to the neutrophil influx [40, 41]. To compare the inflammatory response in skin lesions of mice infected with the WT and the **Δ**RSM strain, we performed skin biopsies for cytokine analysis and histologic examination at six-days post-infection; this time point was chosen as it was the time point with the greatest difference in skin lesion size between the experimental groups. Measurements of interleukin-1 beta (IL-1β), interleukin-6 (IL-6), interleukin-17A (IL-17A), and tumor necrosis factor alpha (TNFα), were obtained as evidence of pro-inflammatory cytokine activity. Cytokine concentrations for all four cytokines measured were significantly greater from mice infected with WT streptococci than mice mock-infected with sterile phosphate-buffered saline (PBS) (Fig 5A). Cytokine concentrations from mice infected with the **Δ**RSM strain were significantly greater than mock-infected or mice infected with the WT strain (Fig 5A). Furthermore, histologic analysis of skin lesions shows predominantly increased neutrophil influx, but also a modest increase in the number of macrophages in the subcutaneous tissue of mice infected with the **Δ**RSM strain compared with WT (Fig 5B). Infiltration of T lymphocytes was not appreciably different between skin lesions of mice infected with WT or the **Δ**RSM strain (Fig 5B). Cytokines IL-6 and IL-17A, in particular, are important for coordinating neutrophil trafficking to areas of infection [42–44]. Our results in mice infected with the **Δ**RSM strain showing enhanced pro-inflammatory cytokine detection, increased neutrophil infiltration, and larger skin lesions, suggests an effect of altered gene transcription patterns in the **Δ**RSM strain and a more robust host inflammatory response compared to mice infected with the WT parent strain. Given the known association of several of the streptococcal gene transcripts down regulated in the **Δ**RSM strain, including *mga*, *emm28*, and *scpA*, with immune evasion properties, we hypothesized that m6A DNA modifications and proper regulation of gene expression are important contributors to immune evasion strategies and/or disruption of host immune responses by *S*. *pyogenes*.

To determine if the loss of specific virulence factors recapitulates the phenotype of the ΔRSM strain in the murine subcutaneous ulcer model, we infected mice with derivatives of strain MEW123 with in-frame deletions of *mga* (strain MEW480, ΔMga), and spectinomycin-resistance cassette disruption mutations of *emm28* (strain 409, Ω*emm28*) and *scpA* (strain 380, Ω*scpA*). As shown in Fig 4C, infection of mice by the ΔMga strain produced skin lesions significantly larger than the WT strain and comparable to the ΔRSM strain in size throughout the experiment. Infection by the Ω*emm28* strain was not statistically different than the WT strain at day 2 and day 4 post-infection; however, by day 6 and day 8 post-infection, the Ω*emm28* strain produced lesions that were statistically significantly larger than the WT (Fig 4C). Infection of mice by the Ω*scpA* strain produced the widest range of murine skin lesion sizes, with some mice having very large lesions following infection (Fig 4C); however, at no time point was the average size of the lesions produced by the Ω*scpA* strain statistically different than WT. Overall, these results suggest that the presence of m6A DNA base modifications produced by M.SpyMEW123 activity correlate with differential transcriptional expression of several *S*. *pyogenes* virulence factors, especially those within the Mga operon, and that these seem to influence host-pathogen interactions at the site of infection.

### Disruption of m6A DNA modifications inhibits streptococcal survival within human neutrophils

**Fig 6:**
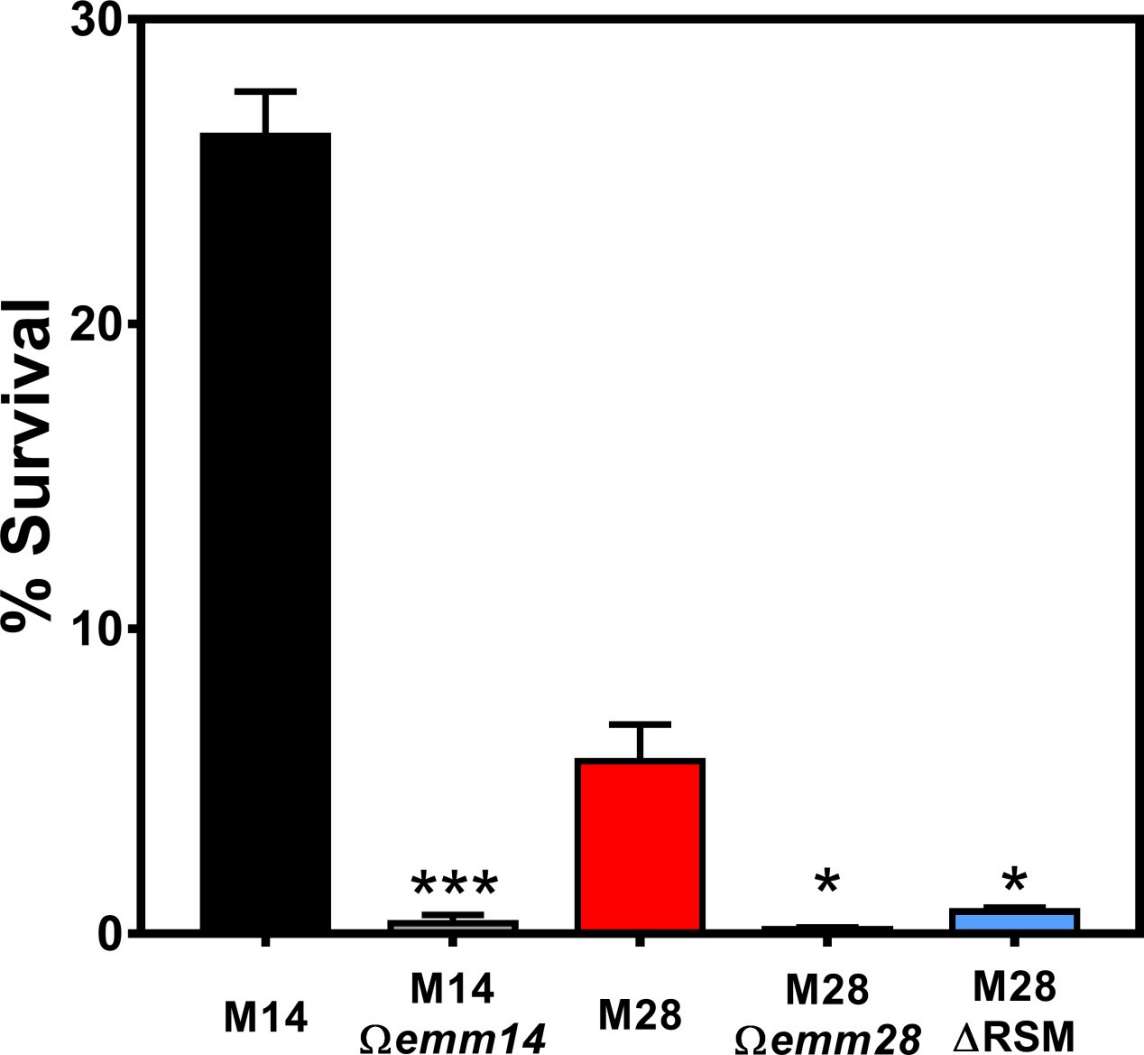
Deletion of MEW123 RSM gene cluster impairs resistance to human neutrophil bactericidal activity. Streptococcal resistance to human neutrophil bactericidal activity was examined using an *in vitro* assay. Human neutrophils were incubated with *S*. *pyogenes* strains in a 1:10 ratio for 10 min. Extracellular streptococci were eliminated with gentamicin and penicillin for 20 min. Neutrophil lysis and release of viable intracellular streptococci was performed with 2% saponin in pH 11 water for 20 min. Surviving CFUs were plated onto THY agar. Shown are streptococcal strains HSC5 (M14) as a positive control, the HSC5 Ω*emm* (M14 Ω*emm*) as a negative control, MEW123 (M28), MEW123 Ω*emm* (M28 Ω*emm*), and the MEW123 RSM deletion mutant (M28 ΔRSM). Shown are mean ± SEM CFU counts performed in triplicate from a representative experiment. An non-paired t-test was used for statistical significance, comparing mutant to parent strain; * *P* < 0.05, *** *P* < 0.001.

A major function of the *S*. *pyogenes* M protein is to promote streptococcal survival, resisting killing by human leukocytes by interfering with bactericidal activity within neutrophils following phagocytosis [45, 46]. Staali *et al*. found that *S*. *pyogenes* strains with or without M protein underwent phagocytosis by neutrophils to similar levels, but only strains expressing M protein survived intracellularly whereas strains lacking M protein expression were rapidly killed [45]. Given our findings that elimination of m6A DNA modifications was associated with decreased transcript expression for *mga* and *emm28*, we wished to compare survival within human neutrophils. Purified human neutrophils were incubated with WT or ΔRSM *S*. *pyogenes* strains using a neutrophil bactericidal assay similar to a previous report [45]. Briefly, streptococci and neutrophils were mixed together allowing the neutrophils to internalize *S*. *pyogenes* strains followed by elimination of extracellular bacteria with penicillin and gentamicin. It was previously determined that there was no significant difference in susceptibility to penicillin and gentamicin at the high concentrations used in these experiments between the WT or ΔRSM strains (S1 Fig). Streptococcus surviving within neutrophils were liberated by treatment with the detergent saponin and plated for viable CFUs. As shown in Fig 6, we utilized serotype M14 HSC5 and a derivative strain with disruption in the M14 *emm* gene (*Ωemm14*) as positive and negative controls, respectively. As expected, the *Ωemm14* mutant was significantly attenuated for intracellular survival within neutrophils compared to the M14 parent strain (Fig 6). Similarly, we compared survival of the MEW123 parent strain (M28) and its cognate strain with disruption of the M28 *emm* gene (*Ωemm28)* or the ΔRSM mutant. We found that both the *Ωemm28* and the ΔRSM mutant were significantly attenuated for intracellular survival compared to the M28 parent strain, further confirming the role of M protein in promoting intracellular neutrophil survival by the serotype M28 MEW123 strain, in addition to demonstrating correlation of m6A DNA base modifications with differential expression of M protein (Fig 6). These results provide further support for m6A DNA base modifications in *S*. *pyogenes* as important for promoting streptococcal virulence, possibly by influencing virulence factor expression.

### Disruption of m6A DNA modifications inhibits adherence to human vaginal epithelial cells *in vitro*, but does not appear to impair carriage *in vivo* in a murine vaginal colonization model

From the RNA-seq results we found that the ΔRSM strain had significantly decreased transcript expression of several recognized and known adhesin proteins, including M28, M-like protein, collagen-binding protein, and fibronectin-binding proteins, as well as several hypothetical surface proteins [38, 47–49]. As a group, serotype M28 *S*. *pyogenes* are overrepresented in cases of human infection within the female urogenital tract, including vulvovaginitis and puerperal sepsis (a.k.a. “childbed fever”) [50–53]. Serotype M28 *S*. *pyogenes* have a particular predilection for cervical and vaginal epithelium due to surface proteins, including protein R28 among others, which may explain the overrepresentation of this serotype with infections in this niche [15, 54]. Therefore, we asked if m6A DNA modifications influenced adherence of the serotype M28 MEW123 strain to human vaginal epithelial cells.

**Fig 7:**
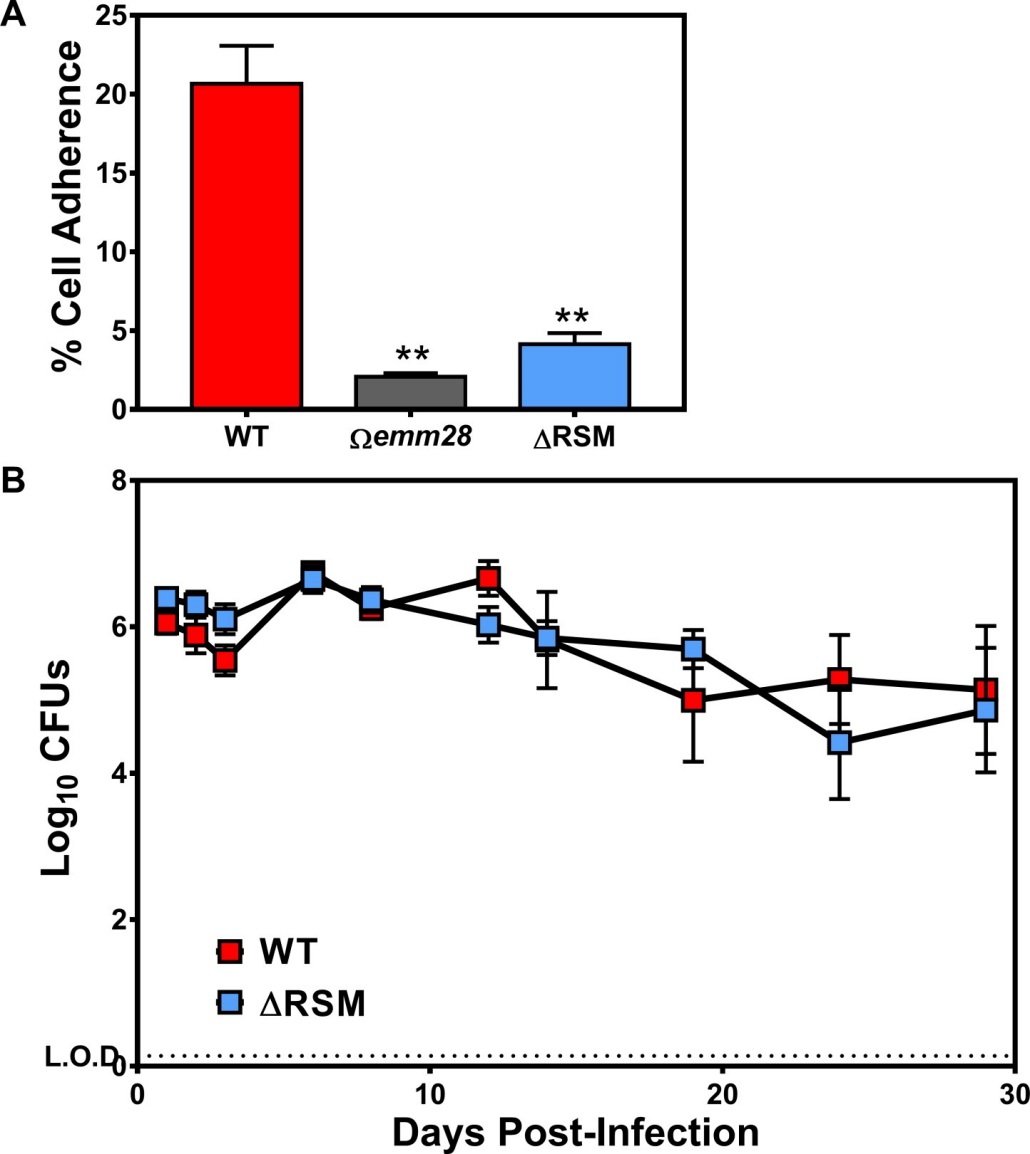
Deletion of MEW123 RSM gene cluster impairs streptococcal adherence to human vaginal epithelial cells, but does not impact carriage duration in a murine vaginal colonization model. A) Confluent wells of VK E6E7 human vaginal epithelial cells were inoculated with 5x10^6^ CFUs of *S*. *pyogenes* MEW123 (WT, red), the *emm28* gene-disrupted mutant (*Ωemm28*, gray), or the RSM deletion mutant (ΔRSM, blue) (multiplicity of infection 5–10:1). Following 1h incubation, non-adherent streptococci were washed away with sterile PBS followed by lysis of the epithelial cells with pH 11 water, serial dilution, and determination of viable streptococci remaining. The % Cell Associated (mostly adherent plus few intracellular) is the percentage of the inoculum CFUs remaining detectable at 1h. Significant differences between groups were calculated by non-paired t-test (** *P* < 0.01, n = 6 replicates per point). B) Estrogenized female C57BL/6J mice were intravaginally inoculated with 1x10^6^ CFU of *S*. *pyogenes* MEW123 (WT, red), or the RSM deletion mutant (ΔRSM, blue). Mice were cultured by intravaginal washes with sterile PBS and plated onto selective media (THY supplemented with streptomycin 1000 μg/mL) for quantification. Shown are mean ± SEM Log_10_ CFU counts over time post-infection of 10 mice per group pooled from two separate experiments. A repeated-measures ANOVA was used for statistical significance; at no time point tested over the 28-day experiment were the two groups statistically significantly different.

As shown in Fig 7A, disruption of m6A DNA modifications in the ΔRSM strain was indeed associated with significantly decreased adherence to human vaginal epithelial cells *in vitro* compared to the WT parent strain. The attenuation in vaginal epithelial cell adherence by the ΔRSM strain was comparable to a strain lacking expression of the M protein (*Ωemm28*), suggesting that decreased expression of M protein, among other adhesins, by the ΔRSM strain was at least partly responsible for decreased adherence (Fig 7A). To determine if impaired adherence to human vaginal cells *in vitro* translated to impaired vaginal mucosal colonization *in vivo*, we utilized a murine vaginal model and compared streptococcal carriage burdens over time [40, 55]. In contrast to the results of the *in vitro* adherence assay, using the murine vaginal carriage model we found no significant difference in vaginal streptococcal burdens in comparison of mice inoculated with either the WT or the ΔRSM strains over the course of the 28-day experiment (Fig 7B). Given that human cells are the natural hosts of *S*. *pyogenes*, this may be an example of the human-restricted nature of *S*. *pyogenes* in which a murine model cannot adequately replicate the natural human environment in which this pathogen evolved to survive. Nevertheless, our overall results showed several key differences in virulence phenotypes correlating with alterations in gene transcription associated with streptococcal m6A DNA methylation.

## Discussion

In this report, we provide evidence that m6A DNA base modifications influence gene transcription patterns and overall virulence properties in a major gram-positive bacterial pathogen of humans, *S*. *pyogenes*. The *S*. *pyogenes* RM system, SpyMEW123I, is a Type I RM system and is responsible for the majority of m6A base modifications distributed throughout the *S*. *pyogenes* genome. The target consensus sequences identified by our study, 5' GCANNNNNTTYG and its corresponding partner motif 5' CRAANNNNNNTGC, were consistent with m6A motifs identified in *S*. *pyogenes* previously reported by Blow *et al* [5]. We found approximately 412 occurrences of each m6A site with the majority found within coding regions. Interestingly, we found that not all m6A sequence motifs were consistently modified to the same extent; only about 70% of consensus sites were modified in at least 75% of sequencing reads, suggesting that m6A modifications may be intermittently present with additional functions beyond simple protection from restriction, including influencing gene expression patterns based on timing of hemi- or full-methylation status. It is not known at this time whether all of the m6A sites, or only the sites within the intergenic regions, would participate in influencing transcriptional expression, but methylation events modifying access of transcriptional regulators to intergenic promoter regions would be a potential mechanism.

With the introduction of SMRT sequencing, groups have now identified m6A DNA modifications within a diversity of prokaryotes, including *E*. *coli*, *Campylobacter jejuni*, *Salmonella enterica* serovar Typhimurium, *Vibrio breoganii*, *Geobacter metallireducens*, *Chromhalobacter salexigens*, *Bacillus cereus*, and *Borrelia burgdorferi* [33–35, 56, 57]. Additional evidence of 5-methylcytosine (m5C) DNA modifications influencing transcriptional expression of multiple genes with an impact on several phenotypic traits has recently been described in *Helicobacter pylori*, further expanding the recognized influence of prokaryotic methylation modifications [58]. Some of the DNA modifications described have been linked to orphan MTases without an associated endonuclease, such as DNA Adenine Methyltransferase (Dam) of *S*. *enterica*, *E*. *coli*, and *Haemophilus influenzae* [56, 59, 60]. Uncoupling DNA methylation from restriction endonuclease protection is conceptually easier to envision with an orphan MTase, freeing the orphan MTase to have roles in DNA mismatch repair and influencing gene expression of potential virulence factors [6]. Indeed, Dam-dependent DNA modifications in *S*. *enterica* have been linked to alterations of gene expression and virulence [56]. However, two examples have recently been reported in *C*. *jejuni* and *B*. *burgdorferi* of intact RM systems also influencing gene expression patterns [34, 35]. Both of the RM systems in these organisms are representatives of Type IIG RM systems, which differ significantly from the Type I RM system described here for *S*. *pyogenes* in that they consist of a single polypeptide with both REase and MTase activity [34, 35, 61]. The effects on gene expression conferred by these systems in *C*. *jejuni* and *B*. *burgforferi* were noted by Casselli *et al*. to be more modest in terms of numbers of genes influenced by m6A base modifications when compared to the larger number of transcriptional changes found from the standalone activity of Dam MTase in Salmonella [34, 35, 56]. It would seem that with an intact RM system the conditions involved in determining gene expression is more stringent and regulates a fewer number of genes than orphan MTases. DNA methylation from Type I RM systems has also been well-established in phase variation in a number of gram-positive pathogens, including *Streptococcus pneumoniae*, *Streptococcus suis*, *Listeria monocytogenes*, and *Mycoplasma pulmonis*, which can have downstream effects on gene expression [62–65]. In phase variation, switching of specificity subunits of Type I RM systems results in cells with different sites of methylation within the population, which can create heterogeneity in gene expression. The role of methylation in phase variation differs from our findings here as we show that loss of methylation at a single site (i.e. not switching of specificity subunits to create methylation at diverse sites) results in the down regulation of a very defined subset of genes.

The M.SpyMEW123I MTase activity we describe here modifies 412 sites in the MEW123 genome, whereas Dam-modified recognition sites approximate 19,000 per chromosome [56]. Perhaps the context of the m6A recognition motif in a particular intergenic promoter region, combined with specific transcription factors sensitive to the presence or absence of m6A modifications, determines the specificity of which genes an intact RM system will influence. Our results reported here demonstrate that the *S*. *pyogenes* Type I RM system is functional as a protective mechanism with restricting uptake of foreign DNA (Fig 2A). Similar results were found by Okada *et al*. in a series of *emm1 S*. *pyogenes* isolates from Japan with spontaneous deletions in their Type I RM systems; isolates lacking the Type I RM system had significantly increased rates of transformation with foreign plasmid DNA [21]. While their study did not specifically address virulence properties of isolates lacking the RM system, the authors speculated that enhanced rates of DNA uptake and transformation exhibited by strains lacking REase activity may be beneficial by allowing uptake of potentially advantageous genes from the environment contributing to overall fitness.

Inactivation of the SpyMEW123I RM system was associated with significant dysregulation of gene transcript expression in broth culture, with 20 genes from at least six separate gene clusters/operons significantly down regulated (Table 4). Notable among the down regulated genes were the trans-acting regulator Mga, the M-like protein, M28 protein, C5a peptidase (ScpA), a cell surface protein, a collagen-like surface protein (SclA), the Serum Opacity Factor (SOF), and a fibronectin-binding protein (SfbX). Most of these genes are regulated by the Mga transcriptional regulator in serotypes that have been investigated. Mga is a ubiquitous stand-alone regulator primarily active during exponential growth phase and is responsible for influencing expression of over 10% of the *S*. *pyogenes* genome, primarily genes involved in metabolism, but also many virulence factors including adhesins and surface proteins involved in immune evasion [37, 38]. Mga binds to upstream promoter regions to activate high-level transcription of genes in the Mga core regulon [66]. The majority of Mga-regulated promoters, including most of the genes in the core Mga regulon, contain a single Mga binding site centered around position -54 and overlapping the -35 region of the gene promoter, likely interacting with the α-subunit of RNA polymerase [67]. In theory, m6A base modifications at or around this site could potentially influence Mga and RNA polymerase binding to the promoter region, perhaps by stabilizing or localizing Mga to the proper site, promoting activation of gene transcription. Consistent with this hypothesis, examination of the genome sequences upstream of the Mga open reading frame for *S*. *pyogenes* strains MEW123, MEW427, and SF370, all reveal the existence of the m6A consensus motifs approximately 800 bp upstream of the *mga* start codon [12, 13, 20]. It is unclear if, or how, this m6A motif site located upstream of the predicted Mga promoter region activates Mga expression. The mechanism of m6A-dependent regulation of the *mga* locus is the subject of active investigation by our group.

Regulation of virulence factor expression in response to different environmental cues and stresses is critical to the success of *S*. *pyogenes* survival and pathogenesis. Over 30 recognized transcriptional regulatory proteins and 13 two-component regulatory systems must function to coordinate virulence factor expression properly [17, 18]. We found that loss of m6A DNA modifications in our ΔRSM mutant correlated with significant changes in virulence properties of *S*. *pyogenes*. In a murine model of subcutaneous ulcer formation, we noted that mice infected with the ΔRSM mutant displayed enhanced inflammatory responses compared to mice infected with the WT strain, with comparatively larger skin lesions, increased detection of pro-inflammatory cytokine levels, and enhanced neutrophil infiltrates on histologic examination (Figs 4 and 5). Disruption of m6A DNA modifications and an associated dysregulation of gene transcript expression may result in failed activation of multiple important adhesins and streptococcal proteins involved in evading host immunity (Fig 3 and Table 4). For example, neutrophilic infiltration in response to bacterial infections is enhanced by activity of host chemotaxins, chiefly complement protein C5a. A major virulence determinant of *S*. *pyogenes* aiding immune evasion is to degrade complement C5a through activity of ScpA, a surface-expressed, serine-protease specifically degrading host C5a and interfering with neutrophil recruitment [68]. We found that the ΔRSM mutant exhibited significantly decreased transcript expression for ScpA which may partly explain a more exaggerated neutrophil response to infection with the ΔRSM mutant strain, resulting in more inflammation and larger skin lesions (Figs 4C and 5). Previous investigation into the contribution of ScpA to host immune responses was performed using a murine air sac model of subcutaneous infection performed by Ji *et al*. [69]; air sacs infected with *S*. *pyogenes* lacking ScpA expression exhibited a significantly enhanced host inflammatory response compared to the WT parent, with a neutrophil predominance analogous to our results. Another report found similar to slightly larger skin lesions in mice infected subcutaneously with *S*. *pyogenes* lacking ScpA compared to WT [70]. The effect of *S*. *pyogenes* virulence factors in murine models is not always similar to activity in the human environment; it is known that ScpA does cleave murine C5a, but at slower rates compared to human C5a, and these differences may impact our ability to detect phenotypes in these non-human systems [71]. Similar to our own results with the Ω*scpA* strain infections, the results reported by Li *et al*. were not statistically significant suggesting that the individual contribution of ScpA in this murine model may be modest, but when the expression of multiple virulence factors is disrupted the effects may be more apparent. Indeed, our experiments in the skin lesion model with the ΔRSM and the Δ*mga* strains showed significant differences in lesion size and inflammatory response compared to the WT and complemented mutant strains. Both of these mutant strains would be expected to have similar patterns of differential gene expression and as result they phenocopy each other in this model. Decreased expression of several adhesins and other factors may have contributed to enhanced spread of the infection together with an exaggerated host inflammatory response resulting in larger areas of inflammation and larger skin lesion formation.

Decreased M protein expression, among other adhesins, also explains the decreased *in vitro* adherence of the ΔRSM mutant to human vaginal epithelial cells. Interestingly, the decreased adherence to human vaginal epithelial cells *in vitro* did not correlate with disrupted carriage in the murine vaginal mucosa colonization model. This suggests that there are additional adhesins not influenced by m6A DNA modifications that are important for promoting and maintaining carriage *in vivo*. One example would be the R28 adhesin of serotype M28 *S*. *pyogenes* strains, which is a major streptococcal adhesin to human cervical epithelial cells [54]. Our RNA-sequencing experiments did not find significant differences in the transcription of the MEW123 R28 gene (AWM59_02815) between WT and the ΔRSM mutant (full data set available in NCBI repository). With only 20 genes significantly downregulated in the ΔRSM mutant clearly not all major *S*. *pyogenes* adhesins and virulence factors are impacted by m6A DNA modifications. Our data show that only a few gene operons, or regulons as in the case of Mga, are differentially expressed in the absence of m6A base modifications in *S*. *pyogenes* and that down regulation of these genes impacts virulence.

In this study, we have demonstrated that the SpyMEW123I RM system and m6A DNA modifications in *S*. *pyogenes* significantly influence DNA restriction activity, in addition to correlating with differential gene transcription and virulence properties of this important human pathogen. Disruption of the SpyMEW123I Type I RM in *S*. *pyogenes* altered the transcriptional profile of the mutant strain resulting in attenuated virulence and impaired evasion of the host immune response in both *in vitro* and *in vivo* models. Similar to our results, disruption of Type IIG RM systems in *C*. *jejuni* and *B*. *burgdorferi* also interfered with genetic regulation of virulence factors of those pathogens [34, 35]. Together, these findings demonstrate that intact RM systems in these bacterial pathogens, and likely many other prokaryotes, can exert multiple functions, including restriction-mediated protection from foreign DNA in addition to influencing gene expression. Understanding how m6A DNA modifications influence virulence properties in these organisms could potentially identify targets for therapeutic intervention, potentially changing patterns of virulence factor expression resulting in strain attenuation helping to prevent human disease. Further investigation is necessary to fully comprehend the many functions of DNA methylation and the complex nature of bacterial physiology and pathogenesis.

## Materials and methods

### Ethics statement

Experimental protocols involving the use of mice were reviewed and approved by the Institutional Animal Care and Use Committee (IACUC) of the University of Michigan Medical School (Ann Arbor, MI, USA). The University of Michigan IACUC complies with the policies and standards as outlined in the Animal Welfare Act and the “Guide for the Care and Use of Laboratory Animals,” [72]. The protocol numbers approved by the University of Michigan IACUC are as follows: Skin and Soft Tissue Infection Model of *Streptococcus pyogenes* Virulence (PRO00007495), and Murine Vaginal Colonization Model for *Streptococcus pyogenes* (PRO00007218). For consistency, all experiments utilized female C57BL/6J mice at approximately 6 weeks of age at the time of use. Mice were purchased from The Jackson Laboratories (catalog #000664) (Bar Harbor, ME, USA), and maintained in a University of Michigan animal facility with biohazard containment properties. Following arrival, mice were allowed to acclimatize in the facility for one week prior to beginning experiments. When manipulated, mice were briefly sedated by inhalation of isoflurane via drop jar dosing. Animals were inspected at least once daily for evidence of suffering, manifested by significantly diminished or no activity, decreased appetite, poor grooming, increased respiratory rate, or weight loss greater than 15% of body weight; if evidence of suffering was identified, then the mouse was euthanized. Euthanasia was primarily through carbon dioxide asphyxiation with a subsequent secondary method of euthanasia, including induction of bilateral pneumothorax, decapitation, and/or removal of a vital organ.

### Bacterial strains, media, and growth conditions

The principal strain used in this study was *S*. *pyogenes* MEW123, a streptomycin-resistant (*rpsL*_K56T_), serotype M28 pharyngeal isolate [55]. Other strains used are listed in Table 1. Growth rates and yields of MEW123 and associated mutant strains were measured using a Synergy HTX plate reader (BioTek, Winooski, VT, USA) in 96 well plates (Greiner Bio-One, Monroe, NC, USA). Briefly, 4μl of overnight culture grown in THY broth was inoculated into 200μl of the described fresh media, with identical strains and conditions measured in at least triplicate. Growth was at 37°C, room air, in static conditions for 12 hours and OD_620nm_ was measured every 3 seconds. Unless otherwise noted, all *S*. *pyogenes* strains had equivalent growth rates and yields under all *in vitro* conditions tested (S1 Fig). Routine culture of *S*. *pyogenes* was performed in Todd-Hewitt medium (Becton Dickinson, Franklin Lakes, NJ, USA) supplemented with 0.2% yeast extract (Difco Laboratories, Detroit, MI, USA) (THY media). Where required, Bacto agar (Difco) was added to a final concentration of 1.4% (w/v) to produce solid media. Gene expression experiments used C-Medium, a lower-glucose, higher-protein media that more closely resembles *in vivo* conditions [73]. Incubation was performed at 37°C under anaerobic conditions (GasPack™, Becton Dickinson) for solid media, or in sealed tubes without agitation for broth media. Aerobic culture was conducted as described [74]. For inoculation of mice, *S*. *pyogenes* was harvested from culture in THY broth at early logarithmic-phase (OD_600_ 0.2), washed once in PBS, briefly sonicated on ice to break up long streptococcal chains, and resuspended in PBS to 10^8^ CFU/mL. Molecular cloning used *Escherichia coli* strain DH5a (Invitrogen, Grand Island, NY, USA) cultured in LB broth. When appropriate, antibiotics were added at the following concentrations: erythromycin, 500 μg/mL for *E*. *coli* and 1 μg/mL for *S*. *pyogenes*; chloramphenicol, 20 μg/mL for *E*. *coli* and 3 μg/mL for *S*. *pyogenes*; spectinomycin, 100 μg/mL for both *E*. *coli* and *S*. *pyogenes*; and streptomycin, 1000 μg/mL for *S*. *pyogenes*. In some experiments, growth was monitored in THY broth supplemented with either penicillin, gentamicin, or erythromycin at concentrations ranging from 0.05 μg/mL to 100 μg/mL. All antibiotics were obtained from Sigma Chemical Co., St. Louis, MO, USA.

### Gene cloning and mutant construction

*Streptococcus pyogenes* MEW123 was used as a source strain for DNA, Genbank CP014139.1 [12]. Bacterial strains and plasmid vectors are listed in Table 1. The primers used for PCR amplification and cloning are listed in Table 5. For cloning and routine DNA Sanger sequencing, the Phusion High-Fidelity DNA Polymerase (New England Biolabs, Inc., Ipswich, MA, USA) was used. For routine endpoint PCR amplification standard Taq DNA Polymerase was used (New England Biolabs, Inc.). Polymerase chain reaction products were digested with indicated restriction enzymes and ligated to pJRS233 or pGCP213 for in-frame deletions, pSPC18 for insertional mutations, or pJoy3 as a plasmid vector for transformation efficiency assays. In-frame deletion mutants and insertional mutants were constructed essentially as described [25, 26], [28], and [27], respectively.

**Table 5:** Primers used in this study.

Primer	Sequence 5'-3' (restriction sites underlined)
MEW123 Del-RSM F1	ATATGAATTCGGTTTTTTGGTAAAAAACTTTTTTAGCA
MEW123 Del-RSM R2	TTTTTTGGTCTTTTTTAATCCCCATTCGACATGATA
MEW123 Del-RSM F3	TGGGGATTAAAAAAGACCAAAAAACACCACAGTAGA
MEW123 Del-RSM R4	ATATAAGCTTTTAATTTAACAAATATTTCTAAAGAAAATGGATTGG
pJoy3 123 RSM F	ACCATTATTGTGAGGAACTGCGTTACCGATCCCTTAAAAG
pJoy3 123 RSM R	ACCGATAGCACCCGCGCATGGATGAGATGATTCTATTTTGATTTATAG
123_7895 F	TAAAACGACGGCCAGTGCCAATCTCTTAGAAACAGGTGAAAG
123_7895 R	ATTCGAGCTCGGTACCCGGGAGATATCATTTTGCGCATAG
M28 Emm Hindlll F	CCCAAGCTTATAAACAGTATTCGCTTAGAAAATTAAAAACAGG
M28 Emm BamHI R	CGCGGATCCGTTAGCTGCTTCGCCTGTTGACGGTAACG
M28 ScpA SalI F	CCCGTCGACCTCAATGCACAATCAGACATTAAAGC
M28 ScpA SacI R	CCCGAGCTCTCAATATCGCCACGTTCAATAAGG
M28 Mga 5’ SalI	CCCGTCGACTGACAATAATGTCACAGAT
M28 Mga 5’ SOE R	TTGTTGGCTAGTAAACAATTTACTTACATGC
M28 Mga 3’ SOE F	GTTTACTAGCCAACAAGCAACATCATCATAGGATTTCAGACG
M28 Mga 3’ BamHI	CGCGGATCCCGCTCTTCGAATACTTTGTT
Emm28 RT-PCR F	CAGACTTAGCAGAAGCAAATAGC
Emm28 RT-PCR R	CAGCTTGTTTAGCCAATTGCTC
Mga RT-PCR F	CTTATCTACCCTCAAACGCCTC
Mga RT-PCR R	CGAATTTGCCTCTCATCTCCTG
ScpA RT-PCR F	CACTGATTTTGATGTGATTGTAGACAA
ScpA RT-PCR R	ATGCAAGTGTCAAACGACGATCT
recA RT-PCR F	ATTGATTGATTCTGGTGCGG
recA RT-PCR R	ATTTACGCATGGCCTGACTC
MEW M13 F	CAGGGTTTTCCCAGTCACGAC
MEW M13 R	GAGCGGATAACAATTTCACACAGG

### a. Construction of an in-frame deletion of *SpyMEW123I* gene cluster

The ΔRSM in-frame deletion allele was cloned by splice overlap extension (SOE) PCR [75]. Corresponding GenBank accession numbers for the MEW123 restriction endonuclease gene *hsdR*, specificity subunit *hsdS*, and the methyltransferase subunit *hsdM*, are AWM59_07895, AWM59_07900, and AWM59_07905, respectively. The upstream region of the gene cluster was PCR amplified using primers MEW123 Del-RSM F1 and MEW123 Del-RSM R2, producing a 1.02 kb amplicon. The downstream region of the gene cluster was PCR amplified using primers MEW123 Del-RSM F3 and MEW123 Del-RSM R4, producing a 1.02 kb amplicon. These two amplicons contain complementary ends that anneal together and essentially will produce an in-frame deletion of the three-gene restriction endonuclease, specificity subunit, and DNA methyltransferase open reading frames. The two amplicons were mixed together as template and further amplified using primers MEW123 Del-RSM F1 and MEW123 Del-RSM R4, the resulting amplicon was approximately 2.04 kb and contained a unique EcoRI site at the 5’ end and a unique HindIII site at the 3' end. The resulting amplicon was digested with EcoRI and HindIII, and inserted within same restriction sites of the *E*. *coli* to *S*. *pyogenes* temperature-sensitive vector for allelic replacements, pGCP213 [26], producing plasmid pKJ24. The pKJ24 plasmid was confirmed by Sanger DNA sequencing using primers MEW M13 F and MEW M13 R, which bind just outside and flank the multiple cloning site region within pGCP213. Electrocompetent cells of MEW123 were prepared and transformation was performed essentially as previously described [76]. The pKJ24 plasmid carrying the RSM in-frame deletion was transformed into electrocompetent *S*. *pyogenes* MEW123 through electroporation with conditions as described above. Erythromycin-resistant transformants were handled according to the temperature-sensitive selection protocol as previously described [26]. Final clones of *S*. *pyogenes* that had successfully replaced the full-length genomic RSM gene cluster with the in-frame deletion allele were screened by endpoint PCR and confirmed by Sanger DNA sequencing. The resulting strain containing the in-frame deletion allele (ΔRSM) was identified as MEW513.

### b. Construction of ΔRSM strain complemented *in trans* with plasmid-encoded RSM operon

GenBank accession numbers for the MEW123 restriction endonuclease gene *hsdR*, specificity subunit gene *hsdS*, and the methyltransferase subunit gene *hsdM*, are AWM59_07895, AWM59_07900, and AWM59_07905, respectively. The operon was cloned by PCR using primers pJoy3_123_RSM_F and pJoy3 123 RSM R, producing an amplicon of approximately 6 kb. This fragment was inserted into plasmid pJoy3 linearized by digestion with EcoRI and SphI using the NEBuilder® HiFi DNA Assembly kit (New England Biolabs, Inc.), producing plasmid pEH01. This plasmid was transformed into electrocompetent *S*. *pyogenes* MEW513 through electroporation with conditions as described above. Chloramphenicol-resistant clones were selected and screened by endpoint PCR, with restoration of m6A methylation activity confirmed by dot blot. The resulting strain containing the plasmid encoded RSM operon for complementation (ΔRSM/pRSM) was identified as strain MEW552.

### c. Construction of spectinomycin-cassette disruption mutant of restriction endonuclease gene, *hsdR*

The GenBank accession number for the restriction-endonuclease subunit gene, *hsdR*, is AWM59_07895. A fragment of the endonuclease open reading frame was cloned by PCR using primers 123_7895 F and 123_7895 R, producing an amplicon of approximately 950 bp. This fragment was inserted into plasmid pSpc18 linearized by digestion with HindIII and BamHI using the NEBuilder® HiFi DNA Assembly kit (New England Biolabs, Inc.), producing plasmid pKJ19. This plasmid was transformed into electrocompetent *S*. *pyogenes* MEW123 through electroporation with conditions as described above. Spectinomycin-resistant clones were selected and screened by endpoint PCR, with final confirmation by Sanger DNA sequencing. The resulting strain containing the spectinomycin-resistance cassette insertion disrupting the restriction endonuclease gene *hsdR* (ΩRE) was identified as strain MEW489.

### d. Construction of in-frame deletion of *mga*

The GenBank accession number for the MEW123 Mga protein, gene *mga*, is AWM59_08335. An in-frame deletion allele of *mga* was cloned by splice-overlap extension (SOE) PCR [75]. The upstream region of the *mga* gene was cloned using primers M28 Mga 5’ SalI and M28 Mga 5’ SOE R, producing an amplicon of approximately 420 bp. The downstream region of the mga gene was cloned using primers M28 Mga 3’ BamHI and M28 Mga 3’ SOE F, producing an amplicon of approximately 410 bp. The two amplicons are mixed together as template and amplified using the outside primers M28 MGA 5’ SalI and M28 Mga 3’ BamHI, producing an amplicon of approximately 830 bp. This amplicon was subsequently digested with BamHI and SalI and ligated into the *E*. *coli* to *S*. *pyogenes* temperature-sensitive vector for allelic replacements, plasmid pJRS233 [25], cut similarly with BamHI and SalI. The resulting plasmid of was named pIL01, with confirmation by Sanger DNA sequencing and PCR verification. Electrocompetent cells of MEW123 were prepared and transformation with plasmid pIL01 was performed essentially as previously described [76]. Erythromycin-resistant transformants were handled according to the temperature-sensitive selection protocol as previously described [26]. Final clones of *S*. *pyogenes* that had successfully replaced the full-length genomic *mga* allele with the in-frame deletion allele were screened by endpoint PCR and confirmed by Sanger DNA sequencing. The resulting strain containing the in-frame deletion allele (Δ*mga*) was identified as MEW480.

### e. Construction of spectinomycin-cassette disruption mutant of strain MEW123 *scpA* gene (ScpA protein)

The GenBank accession number for the MEW123 *scpA* gene is AWM59_08315. A fragment of the *scpA* open reading frame was cloned by PCR using primers M28 ScpA SalI F and M28 ScpA SacI R, producing an amplicon of approximately 1.1 kb. The amplicon was digested with SalI and SacI and ligated into plasmid pSpc18 linearized with SalI and SacI, producing plasmid pIL09. This plasmid was transformed into electrocompetent *S*. *pyogenes* MEW123 through electroporation with conditions as described above. Spectinomycin-resistant clones were selected and screened by endpoint PCR, with final confirmation by Sanger DNA sequencing. The resulting strain containing the spectinomycin-resistance cassette insertion disrupting the *scpA* gene (*ΩscpA*) was identified as strain MEW380.

### f. Construction of spectinomycin-cassette disruption mutant of strain MEW123 *emm28* gene (M28 protein)

The GenBank accession number for the M28 protein, gene *emm28*, is AWM59_08325. A fragment of the *emm28* open reading frame was cloned by PCR using primers M28 Emm Hindlll F and M28 Emm BamHl R, producing an amplicon of approximately 1.1 kb. This amplicon incorporated unique sites for HindIII and BamHI, and the amplicon was accordingly restriction digested and ligated into plasmid pSpc18 opened with HindIII and BamHI, producing plasmid pIL03. This plasmid was transformed into electrocompetent *S*. *pyogenes* MEW123 through electroporation with conditions as described above. Spectinomycin-resistant clones were selected and screened by endpoint PCR, with final confirmation by Sanger DNA sequencing. The resulting strain containing the spectinomycin-resistance cassette insertion disrupting the *emm28* gene (*Ωemm28*) was identified as strain MEW409.

### Transformation efficiency assay

Transformation efficiency was assessed by electroporation of electrocompetent *S*. *pyogenes* strains with 0.5 μg plasmid pJoy3 conferring chloramphenicol resistance isolated from *E*. *coli* DH5α. Electroporation was performed using a Gene Pulser II system (BioRad, Hercules, CA, USA) under the following settings; Volts at 1.75 kV, capacitance at 400Ω, and resistance at 25 μf. Transformants were plated onto THY agar supplemented with chloramphenicol. In addition, a separate aliquot of the sample was plated onto THY agar with no antibiotics to determine the total viable cell count. Transformation efficiency was determined as the number of chloramphenicol resistant cells per total viable cell count.

### SMRT sequencing

Genomic DNA was purified from *S*. *pyogenes* strains MEW123 (WT) and MEW513 (ΔRSM) using the Wizard Genomic DNA Purification Kit (Promega, Madison, WI). Genomic DNA preparation, library preparation, and sequencing of MEW123 was performed as previously described [12]. For MEW513, one Single Molecule Real-Time (SMRT) cell was used to sequence the library prepared with 5 kb mean insert size on the Pacific Biosciences RSII sequencer by the University of Michigan Sequencing Core (https://brcf.medicine.umich.edu/cores/dna-sequencing). Modification and motif analysis was performed using RS_Modification_and_Motif_Analysis.1 version 2.3.0 using the published MEW123 reference genome with an average reference coverage of 501 and 539 for MEW123 and MEW513, respectively. Data generated in this analysis have been deposited in NCBI’s Gene Expression Omnibus [77] and are accessible through GEO Series accession number GSE130428 (https://www.ncbi.nlm.nih.gov/geo/query/acc.cgi?acc=GSE130428).

### Dot blot assay for m6A modification

Genomic DNA was isolated from *S*. *pyogenes* strains MEW123 (WT) and MEW513 (ΔRSM), as described above. DNA was treated with RNAse during purification to remove any contaminating mRNA or rRNA potentially containing m6A base modifications. DNA was denatured by heating at 98°C for 10 min and then placed immediately on ice for 5 minutes. Denatured DNA or unmodified oligonucleotides as a negative control was then spotted at 500 ng per spot onto nitrocellulose membranes and allowed to air dry. Membranes were then placed onto Whatman paper soaked with PBS containing 0.5% Tween 20 (PBST), and DNA was cross-linked to the membranes using a Bio-Rad GS Genelinker using two 125 mJ delivery cycles. Membranes were blocked in 5% milk protein in PBS for 1 h at room temperature and then incubated with a dilution of anti-m6A primary rabbit antibody (2 μg/mL) (EMD Millipore ABE572 Anti-N6-methyladenosine (m6A) Antibody) in 5% milk PBS overnight at 4°C. Primary antibody was removed and the membrane was washed three times with PBST for 5 minutes each wash. The membrane was then incubated with a 1:5,000 dilution of horseradish peroxidase-conjugated anti-rabbit secondary antibody in 5% milk PBS at room temperature for 1 hour. The secondary antibody was removed, and the membrane washed with PBST three times for 5 minutes each wash. Chemiluminescent substrate (Pierce SuperSignal West Femto HRP Substrate, ThermoFisher Scientific, Waltham, MA) was applied and the membrane was visualized.

### RNA-sequencing

Streptococcus from fresh overnight growth on THY agar plates was inoculated into 40 mL of C-media broth and grown statically to mid-log phase OD_600nm_ of 0.6. RNA was then purified using the RiboPure RNA Purification Kit (Life Technologies), for bacteria according to the manufacturer’s recommendations. The University of Michigan Sequencing Core performed ribosomal rRNA depletion using the Ribo-Zero Magnetic Kit, bacteria and subsequent library preparation. Fifty-base single end reads were sequenced on the Illumina HiSeq 4000. Sequence alignment was performed using the Burrows-Wheeler Aligner (BWA) version 0.7.8-r455 to the MEW123 reference genome [12]. Subsequent differential expression analysis was performed using the limma package in R [78]. Differentially expressed genes were called as those that had a Benjamini-Hochberg adjusted p-value less than 0.05 and a log_2_ fold change greater than 1. Log_2_ CPM values were computed using edgeR and were subsequently used to construct the heatmap using the *aheatmap* function as part of the NMF package in R [79, 80]. Data generated in this analysis have been deposited in NCBI’s Gene Expression Omnibus [77] and are accessible through GEO Series accession number GSE130427 (https://www.ncbi.nlm.nih.gov/geo/query/acc.cgi?acc=GSE130427).

### Real-time PCR for comparison of transcript expression

Based on results of the most significantly differentially expressed genes between WT (MEW123) and ΔRSM (MEW513), three genes were selected for independent reverse-transcription cDNA preparation and real-time PCR amplification for relative comparison of transcript expression; *mga*, *emm*28, and *scpA*. *RNA* was isolated as described above from strains grown in C-media broth to mid-log phase OD_600nm_ of 0.6. Synthesis of cDNA was performed using the iScript™ cDNA Synthesis Kit (BioRad). Real time amplification of select genes was performed using an iCycler Thermocycler (BioRad) and iQ SYBR Green Supermix (BioRad). Sequences for RT-PCR primers are as shown in Table 5. Relative transcript levels were determined using the *recA* transcript as reference by the 2^(-ΔΔCt)^ method [81]. All RNA was stored at -80ºC. All cDNA was stored at -20ºC or utilized directly for comparative RT-PCR analysis. For each experiment, three biological replicates were analyzed in duplicate. Statistical significance was examined using the paired t-test in Prism 6 (GraphPad).

### Murine subcutaneous infection model

Inflammatory infection of murine subcutaneous tissue was conducted as described in detail [41]. On the day of infection, mice sedated by inhalation of isoflurane received a subcutaneous injection of 100 μl PBS containing 1 x 10^7^ *S*. *pyogenes* into the shaved flank. Following infection, the resulting ulcers were photographed over several days and the areas of the irregular lesions were calculated using ImageJ software as described in detail elsewhere [82, 83]. Skin biopsies were obtained from euthanized mice and homogenized in 1 mL ice cold PBS using a FastPrep-24 homogenizer (MP Biomedicals, LLC., Santa Ana, CA); tissue was homogenized in 2 mL conical screw top vials with 3.2 mm stainless steel beads (Fisher Scientific, Pittsburg, PA) with two FastPrep cycles of speed 6.0 for 45 sec, with a 5 min ice incubation between pulses to prevent overheating.

### Murine tissue cytokine analysis

Samples of mouse skin and subcutaneous tissue homogenates were harvested at six-days post-infection. Cytokine protein concentrations were determined by a multiplex murine ELISA assay (EMD Millipore, Billerica, MA, USA) according to the manufacturer's protocol.

### Murine tissue histologic analysis

Murine skin biopsies were obtained at six-days post-infection and were fixed in 4% formalin and dehydrated up to 70% ethanol prior to paraffin embedding through the University of Michigan Pathology Core for Animal Research (PCAR). H&E staining and immunohistochemistry services were performed by the PCAR using commercially available anti-CD3 (T lymphocytes), and anti-F4/80 (macrophages) antibodies (Abcam, Cambridge, MA, USA). Digital images were obtained with an EC3 digital imaging system (Leica Microsystems, Buffalo Grove, IL, USA) using Leica Acquisition Software (Leica Microsystems). Adjustments to contrast in digital images were applied equally to all experimental and control images.

### Human vaginal epithelial cell *in vitro* assays

Adherence of *S*. *pyogenes* strains was assessed to an established human vaginal epithelial cell line, VK2/E6E7, using methods similar to those previously described [84–86]. The human vaginal epithelial cell line VK2/E6E7 was purchased from the American Type Culture Collection (ATCC, Manassas, Virginia), and cells were grown and maintained in media and conditions as recommended by ATCC. Human cells were grown to confluence in 12-well tissue culture-treated plates and washed with sterile PBS prior to inoculation with bacteria. *S*. *pyogenes* strains were grown in THY broth to early stationary phase (OD_600nm_ 0.6), washed twice in sterile PBS, and adjusted to give an inoculum of ~5 x 10^6^ CFU in 1 mL per well, for a multiplicity of infection (MOI) of ~5. Bacteria and human cells were incubated at 37°C in 5% carbon dioxide for 60 min, after which time the supernatants were removed and cells were washed four times with 2 mL sterile PBS to remove non-adherent organisms. To recover *S*. *pyogenes* from the epithelial cells, each well was treated with 0.2 mL 0.25% Trypsin-EDTA (Invitrogen) and incubated at 37°C for 5 min, and then lysed by addition of 0.8 mL sterile water at pH 11. Lysis in water at pH 11 was shown to result in a more complete eukaryotic cellular breakdown with maximal recovery of bacteria from the surface in addition to intracellular reservoirs [87]. This method recovers all cell-associated streptococci, predominantly extracellular adherent cells with a relatively smaller amount of intracellular cells. The cell suspension was serially diluted in PBS and plated onto THY agar for determination of viable CFU count. The total cell-associated CFU percentage was calculated as (total CFU recovered from the well/CFU of the original input inoculum) x 100%.

### Murine vaginal colonization model

Experiments were performed as previously described [55]. To synchronize estral cycles, sedated mice were estrogen supplemented by intra-peritoneal injection with 0.5 mg β-estradiol 17-valerate (Sigma) dissolved in 0.1 mL sterile sesame oil (Sigma) 2 days prior to streptococcal inoculation and again on the day of inoculation (considered day #0). On day #0, sedated mice were inoculated with ~1 x 10^6^ colony forming units (CFUs) instilled into the vaginal vault using a P20 micropipetter (Gilson, Inc., Middleton, WI) in a total volume of 20 μL PBS. At successive intervals over a 1-month period post-inoculation, the vaginal vaults of sedated mice were gently washed with 50 μL sterile PBS and serial dilutions in sterile PBS were plated onto THY agar plates supplemented with 1000 μg/mL streptomycin to determine viable CFUs. This concentration of streptomycin suppressed growth of normal mouse vaginal flora but had no effect on the plating efficiency of the streptomycin-resistant *S*. *pyogenes* strains. For colonization experiments, between 5 to 20 mice were tested per *S*. *pyogenes* strain, as indicated in the relevant figure legends.

### Human neutrophil bactericidal activity assay

Human neutrophils were purchased from a commercial supplier (Astarte Biologics, Bothell, WA, USA) and prepared according to supplier recommendations to a concentration of 5 x 10^6^ cells/mL in room temperature Hank’s Balanced Salt Solution (HBSS). Neutrophil bactericidal assay was performed similar to that reported by Staali *et al*. [45]. Briefly, streptococcal strains were grown in fresh C-media to mid-log phase (OD_600nm_ of 0.6) and were washed twice in HBSS with calcium and magnesium, but without Phenol Red (Sigma, St. Louis, MO, USA). Streptococci were counted using a hemocytometer and adjusted to a concentration of 5 x 10^7^ CFU/mL in room temperature HBSS. Neutrophils and streptococci were mixed in a 1:10 ratio of neutrophils to bacteria, and were incubated together for 10 minutes at 37°C. Next, extracellular streptococci were eliminated by addition of gentamicin (100 μg/mL) and penicillin (5 μg/mL) in HBSS for 20 minutes at 37°C. Next, cells were diluted in 1 mL of HBSS, centrifuged at 400g x 5 min, and washed with 1 mL fresh HBSS. The wash was repeated a second time in HBSS and the final cell pellet was resuspended in 50 μL of 2% saponin in distilled water at pH 11 and allowed to remain at room temperature for 20 minutes to lyse neutrophils and release viable intracellular streptococci. The cells were diluted in distilled pH 11 water and aliquots plated onto fresh THY agar media for CFU counts. Three biological replicate experiments for each strain were performed.

### Statistical analyses

Comparison of nonparametric data sets was performed using the Mann-Whitney U-test to determine significant differences. Differences between groups for recovery of CFU in vaginal washes were tested using a repeated measures analysis of variance. Differences in relative transcript levels were tested for significance with a two-tailed paired t-test. Differences in VK cell adherence and in neutrophil bactericidal survival assays were compared using a non-paired t-test. For all tests, the null hypothesis was rejected for *P* < 0.05. Computation utilized the resources available in GraphPad Prism™ (GraphPad Software, Inc., San Diego, CA).

## Supporting information

S1 Fig Growth curves of WT and ΔRSM mutant are similar in THY broth and C-media, and in THY-broth containing penicillin, gentamicin, or erythromycin. Growth was monitored using a Synergy HTX plate reader (BioTek) in 96 well plates (Greiner Bio-One). Briefly, 4μl of overnight culture grown in THY broth was inoculated into 200μl of the described fresh media, with identical strains and conditions measured in at least triplicate. Growth was at 37°C, room air, in static conditions for 12 hours (time on X-axis) and OD_620nm_ was measured every 3 seconds (Y-axis). Where indicated, antibiotic was added to THY broth at concentrations ranging from 0.05 μg/mL to 100 μg/mL, with concentrations shown in the color-coded key on the right [μg/mL].
(TIF)

S2 Fig Streptococcal CFU counts in skin lesions at day 4 post-infection. Skin lesions from infected mice were dissected on day#4 post-infection and homogenized in 1 mL sterile PBS. The homogenate was serially diluted in sterile PBS and plated onto THY agar plates containing streptomycin (1000 μg/mL) to select for *S*. *pyogenes* strain MEW123 and its mutants. Each point represents the CFU counts from one mouse lesion, with black bars indicating mean CFU values for that group. Groups are MEW123 (WT), MEW513 (ΔRSM), MEW552 (ΔRSM/pRSM), and MEW480 (Δ*mga*). The Mann-Whitney U-test was used to test for statistical significance, though none were statistically different from the WT strain.
(TIF)
