# Supplementary figures and images for: DNA methylation from a Type I restriction modification system influences gene expression and virulence in Streptococcus pyogenes
Source: PLoS Pathog. 2019 Jun 17;15(6):e1007841. doi: 10.1371/journal.ppat.1007841 (PMC6597129; doi:10.1371/journal.ppat.1007841)

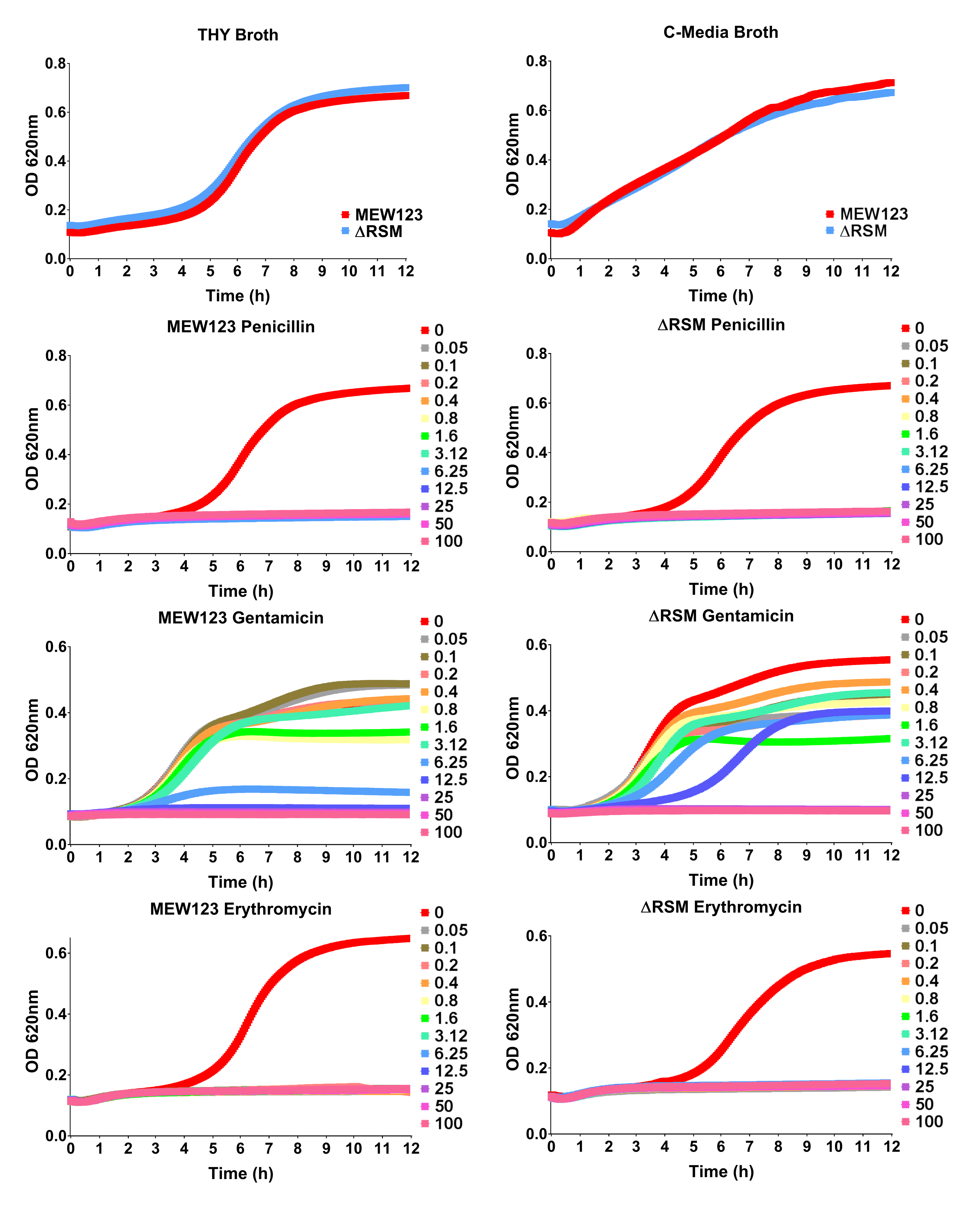

Supplement: S1 Fig — Growth was monitored using a Synergy HTX plate reader (BioTek) in 96 well plates (Greiner Bio-One). Briefly, 4μl of overnight culture grown in THY broth was inoculated into 200μl of the described fresh media, with identical strains and conditions measured in at least triplicate. Growth was at 37°C, room air, in static conditions for 12 hours (time on X-axis) and OD620nm was measured every 3 seconds (Y-axis). Where indicated, antibiotic was added to THY broth at concentrations ranging from 0.05 μg/mL to 100 μg/mL, with concentrations shown in the color-coded key on the right [μg/mL]. (TIF) [file ppat.1007841.s001.tif]

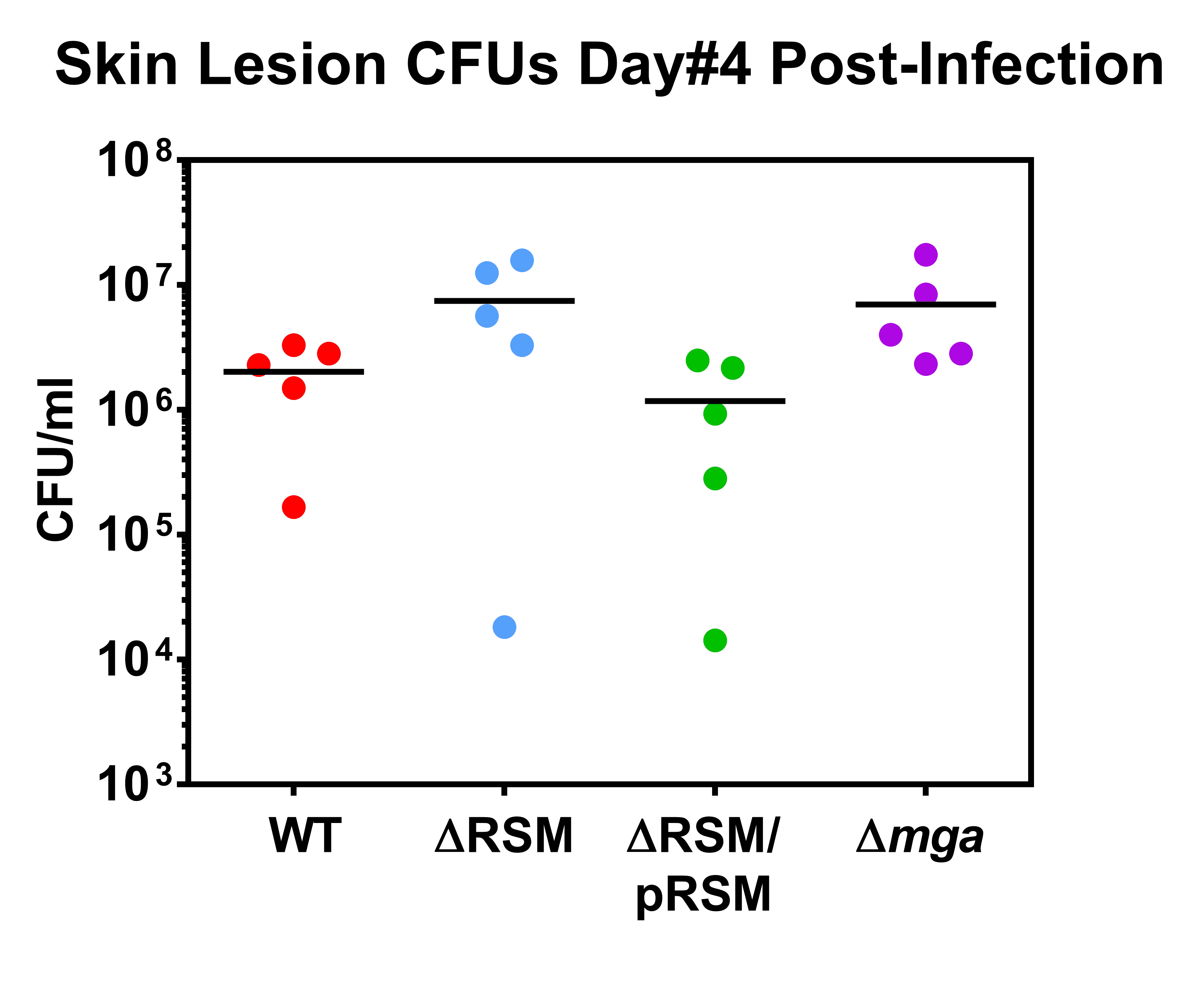

Supplement: S2 Fig — Skin lesions from infected mice were dissected on day#4 post-infection and homogenized in 1 mL sterile PBS. The homogenate was serially diluted in sterile PBS and plated onto THY agar plates containing streptomycin (1000 μg/mL) to select for S. pyogenes strain MEW123 and its mutants. Each point represents the CFU counts from one mouse lesion, with black bars indicating mean CFU values for that group. Groups are MEW123 (WT), MEW513 (ΔRSM), MEW552 (ΔRSM/pRSM), and MEW480 (Δmga). The Mann-Whitney U-test was used to test for statistical significance, though none were statistically different from the WT strain. (TIF) [file ppat.1007841.s002.tif]
